# Adiponectin receptor agonist ameliorates cardiac lipotoxicity via enhancing ceramide metabolism in type 2 diabetic mice

**DOI:** 10.1038/s41419-022-04726-8

**Published:** 2022-03-30

**Authors:** Yaeni Kim, Ji Hee Lim, Eun Nim Kim, Yu Ah Hong, Hun-Jun Park, Sungjin Chung, Bum Soon Choi, Yong-Soo Kim, Ji Yong Park, Hye Won Kim, Cheol Whee Park

**Affiliations:** 1 Department of Internal Medicine, College of Medicine, The Catholic University of Korea, Seoul, Republic of Korea; 2 Transplant Research Center, Convergent Research Consortium for Immunologic Disease, Seoul St. Mary’s Hospital, College of Medicine, The Catholic University of Korea, Seoul, Republic of Korea; 3 Institute for Aging and Metabolic Diseases, College of Medicine, The Catholic University of Korea, Seoul, Republic of Korea; 4 Department of Psychology, Korea University, Seoul, Korea; 5 Department of Rehabilitation, College of Medicine, The Catholic University of Korea, Seoul, Republic of Korea

**Keywords:** Chronic inflammation, DNA metabolism

## Abstract

Accumulation of lipids and their metabolites induces lipotoxicity in diabetic cardiomyopathy. Lowering ceramide concentration could reduce the impact of metabolic damage to target organs. Adiponectin improves lipotoxicity through its receptors (AdiopRs), which have sequence homology with ceramidase enzymes. Therefore, cardioprotective role of AdipoR agonism by AdipoRon was investigated. Sixteen-week-old male *db/m* and *db/db* mice were fed a diet containing AdipoRon for four weeks. Phenotypic and metabolic profiles with associated cellular signaling pathways involved in lipid metabolism were investigated in the mice heart and human cardiomyocytes to establish treatment effect of AdipoRon. AdipoRon ameliorated insulin resistance, fibrosis, M1-dominant inflammation, and apoptosis in association with reduced accumulations of free fatty acid, triglycerides, and TLR4-related ceramide in the heart. This resulted in overall reduction in the level of oxidative stress which ameliorated cardiac hypertrophy and its function. AdipoRon increased the expression of AdipoR1 and AdipoR2 via pAMPK/FoxO1-induced Akt phosphorylation resulting from a decrease in PP2A level. It also increased acid ceramidase activity which reduced ceramide and increased sphingosine-1 phosphate levels in the heart of *db/db* mice and cultured human cardiomyocytes. Consistent upregulation of AdipoRs and their downstream regulatory pathways involving pAMPK/PPARα/PGC-1α levels led to lipid metabolism enhancement, thereby improving lipotoxicity-induced peroxisome biogenesis and oxidative stress. AdipoRon might control oxidative stress, inflammation, and apoptosis in the heart through increased AdipoR expression, acid ceramidase activity, and activation of AMPK-PPARα/PGC-1α and related downstream pathways, collectively improving cardiac lipid metabolism, hypertrophy, and functional parameters.

## Introduction

There is an exponential growth in the incidence of diabetes and obesity-related diseases as a greater proportion of the population is exposed to a state of excess energy caused by increased dietary intake and a sedentary lifestyle. When the energy storage capacity of white adipose tissues becomes saturated, excess fat enters alternative non-oxidative pathways and is redirected towards complex lipid synthesis and accumulation in non-adipose tissues that promote lipotoxicity in the target organs [1].

The heart uses lipids not only to maintain its cellular structure but also as a fuel for cardiac muscle contraction. In diabetes, higher levels of circulating free fatty acids (FFA) in the plasma drive an FFA uptake in the heart higher than the FFA requirement for energy production [2]. This surplus FFA accumulation in the organ enters pathways that use more fatty acids, and conversely, less glucose. The resulting diabetic cardiomyopathy is distinctive in nature, where heart failure is not caused by ischemic injury or hypertension-induced concentric hypertrophy but is rather characterized by the accumulation of such neutral lipids as diacylglycerols, triglycerides, and ceramides in or around the myocardium of non-ischemic heart [3, 4]. Enhanced ceramide biosynthesis is an alternative route to metabolize these excess FFAs, increasing ceramide levels in the myocardium, which directly correlates with the degree of cardiomyocyte apoptosis and cardiac dysfunction in obese diabetic hearts [5, 6].

Ceramides involve a family of lipids consisting of sphingosine covalently linked to a fatty acid that regulates pleiotropic cellular events, including cell differentiation, proliferation, and apoptosis, apart from their established role in maintaining plasma membrane and lipoprotein structures [7, 8]. Ceramides could be produced primarily by *de novo* synthesis or plasma membrane sphingomyelin hydrolysis and the salvage pathway [7]. While the aberrant accumulation of certain ceramide subtypes has been implicated in the development of metabolic disorders, sphingosine-1 phosphate (S1P), generated during ceramidase activation leading to ceramide-to-sphingosine conversion, is a pro-surviving factor that facilitates cellular proliferation and inhibits cell death [9, 10].

Adiponectin is an adipocyte-derived adipokine that exerts pro-metabolic effects by improving systemic insulin sensitivity through the modulation of glucose and lipid homeostasis in the target organs. Its favorable metabolic effects are conferred by binding to its receptors, adiponectin receptor 1 (AdipoR1) and AdipoR2, which in turn activate their primary downstream target molecules, 5’ adenosine monophosphate-activated protein kinase (AMPK) and peroxisome proliferator-activated receptor α (PPARα), respectively [11, 12]. Adiponectin can also modify the effects of toxic ceramide accumulation by increasing ceramidase activity. Ceramidase hydrolyzes ceramide to form sphingosine and subsequently S1P, leading to an increase in the S1P: ceramide ratio. This would otherwise have disrupted the association between inhibitor 2 of protein phosphatase 2 A (I2PP2A) and protein phosphatase 2 A (PP2A), leading to protein kinase B (Akt/PKB) dephosphorylation, which in turn would activate catabolic pathways leading to a net decrease in cellular growth and stability [13, 14].

In this context, the role of sphingolipids and their precursor ceramide, as a regulator of cardiac energy metabolism and signaling, is implicated in obesity and diabetes-associated cardiac dysfunction pathogenesis. Here, we aimed to determine cardioprotective effect of AdipoR agonism, using AdipoRon, by demonstrating possible associated links and altered levels of ceramides and their metabolites with changes in cellular oxidative stress, inflammation, and survival under lipotoxic conditions.

## Methods

### Animal experiments

Six-week-old male C57BLKS/J *db/m* and *db/db* mice were purchased from Jackson Laboratories (BarHarbor, ME). Male C57BLKS/J *db/m* and *db/db* mice were randomly divided into four groups, and received either a regular diet chow or a diet containing AdipoRon. AdipoRon (30 mg/kg; Sigma, St Louis, MO) was mixed into the standard chow diet and provided to *db/db* mice (*db/db* + AdipoR, *n* = 8) and age- and sex-matched *db/m* mice (*db/m* + AdipoR, *n* = 8) from 16 weeks of age for four weeks. Control *db/db* (*db/db* Cont, *n* = 8) and *db/m* mice (*db/m* Cont, *n* = 8) were fed normal diet chow. At week 20, all animals were anesthetized by intraperitoneal injection of 30 mg/kg tiletamine plus zolazepam (Zoletil; Virbac, Carros, France) and 10 mg/kg xylazine hydrochloride (Rompun; Bayer, Leverkusen, Germany) to compare the effect of AdipoRon treatment. Blood was collected from the left ventricle and the plasma was stored at −70 °C for subsequent analyses. Blood glucose levels were measured using an Accu-heck meter (Roche Diagnostics, St Louis, MO) and glycosylated hemoglobin (HbA1c) was determined using an autoanalyzer (Bayer Healthcare, LLC, IN). Plasma insulin concentrations were measured by RIA (Alpco), and the HOMA-IR index was calculated as follows: fasting glucose (mmol/L) × fasting insulin (mU/L)/22.5 [15]. The concentration of serum adiponectin was determined by ELISA (Biosource, Camarillo, CA). A 24-hour urine collection was obtained using metabolic cages at week 20 and urinary albumin concentration was measured by an immunoassay (Bayer, Elkhart, IN). Plasma and urine creatinine concentrations were measured by an enzymatic creatinine assay (Samkwang Medical Laboratory, Seoul, Korea). All animal experiments were performed in accordance with the Laboratory Animals Welfare Act, Guide for the Care and Use of Laboratory Animals, and approved by the Institutional Animal Care and Use Committee (IACUC) at the College of Medicine, Catholic University of Korea (CUMC-2017–0251–01).

### Cell culture and small interfering RNA transfection

Human cardiomyocyte (HCM) (PromoCell GmbH, Heidelberg, Germany) were cultured in Myocyte Growth Medium (PromoCell GmbH, Heidelberg, Germany) at 37 °C in a humidified, 5% CO_2_/95% air atmosphere. Passage 3–9 HCMs were used in the experiment. Thereafter, HCMs were then exposed to low glucose (5 mmol/L D-glucose) or high glucose (HG; 35 mmol/L D-glucose) plus palmitate acid (PA; 500 µM, Sigma-Aldrich), with or without the additional 48-hour application of AdipoRon (5, 10 nM). The sequences of the siRNAs were as follows: AdipoR1, 5ʹ-GGACAACGACUAUCUGCUACATT-3ʹ; AdipoR2, 5ʹ- CCAACUGGAUGGUACACGA-3ʹ; and nonspecific scrambled siRNA, 5ʹ-CCUACGCCACCAAUUUCGU-3ʹ (Bioneer, Daejeon, Korea). Human cardiomyocytes in six-well plates were transfected with a final concentration of 50 nM AdipoR1 and AdipoR2 siRNAs using Lipofectamine2000 in Opti-MEM(R) I reduced serum medium (Gibco Invitrogen, Carlsbad, CA) for 24 h and then the medium was changed back to growth medium for additional incubation. After transfection, cells were treated with AdipoRon (50 nM) in high glucose plus palmitate acid media to evaluate the effects of siRNAs on myocyte reactions.

### Assessment of cardiac functional parameters

Echocardiographic analysis using Hewlett-Packard Sonus 4500 ultrasound machine (Agilent Technologies, Edmonton, Alberta, Canada) was performed as previously described [16] while the animals were anesthetized. Systolic blood pressure was measured with the tail cuff in untrained conscious mice by using the Visitech BP-2000 system (Visitech Systems, Apex, NC). Blood pressures were measured 10 times per day for 4 consecutive days, and a mean value was generated for each mouse.

### Assessment of oxidative stress, intracardiac lipids, and inflammation

To evaluate oxidative stress, we measured the 24-hour urinary 8-hydroxy-deoxyguanosine (8-OHdG; OXIS Health Products, Inc., Portland,OR), and 8-epi-PG F2a (OXISHealth Products, Inc., Portland, OR). The heart lipids were extracted by the method of Bligh and Dyer with slight modifications as previously described [17]. Total cholesterol and triglyceride concentrations were measured by an autoanalyzer (Hitachi 917, Tokyo, Japan) using commercial kits (Wako, Osaka, Japan). Nonesterified fatty acid levels were measured with a JCA-BM1250 automatic analyzer (JEOL, Tokyo, Japan). In addition, the concentrations of inflammatory markers, including monocyte chemoattractant protein (MCP)-1, tumor necrosis factor (TNF)-α, were measured in the tissue lysate or the sera using a commercial ELISA kit purchased from Abcam Inc. (Cambridge, UK). The color generated was determined by measuring the optical density at 450 nm, using a spectrophotometric microtiter plate reader (Molecular Devices Corp., Sunnyvale, CA).

### Measurement of acid ceramidase, SIP, PP2A, and ceramide species

The concentration of intracardiac and cardiomyocyte acid ceramidase concentrations (USBiological, MA, USA) and S1P concentrations (MyBioSource, San Diego, CA, USA) were determined using an ELISA kit. PP2A activity was evaluated after immunoprecipitation of the catalytic (C) subunit of PP2A using a malachite green phosphatase assay kit (Millipore, Billerica, MA, USA). We also measured intracardiac ceramide species with authentic standards (Sigma-Aldrich, St. Louis, MO, USA, Avanti Polar Lipids, Alabaster, AL), ceramide-nonhydroxy fatty acid conjugated to sphingosine (CNS), and ceramide-nonhydroxy fatty acid conjugated to dihydrosphingosine (CNDS) employing UPLC-MS/MC according to the methods previously described [18].

### Histological analysis

Heart samples were emersion fixed in 10% neutral buffered formalin and then paraffin-embedded. Histological analysis was performed on 5-μm paraffin sections (Leica Microsystems), stained with Masson’s trichrome, and examined under bright-field illumination (Olympus BX50; Olympus Optical, Tokyo, Japan). We performed immunohistochemistry for transforming growth factor (TGF)-β1, type IV collagen (Col IV). Cardiac tissue sections were incubated overnight with anti–TGF-β1 (R&D Systems, Minneapolis, MN), anti-Col IV (Biodesign International, Saco,ME) in a humidified chamber at 4 °C. The antibodies were localized using a peroxidase- conjugated secondary antibody and the Vector Impress kit (Vector Laboratories, Burlingame, CA) with a 3,3-diamninobenzidine substrate solution with nickel chloride enhancement. The sections were then dehydrated in ethanol, cleared in xylene, and mounted without counterstaining. The proportion of apoptotic cells were also determined using *ApopTaq In Situ Apoptosis Detection Kits* (EMD Millipore, Temecula, USA), based on a terminal deoxynucleotidyl transferase-mediated dUTP nick-end labeling (TUNEL) assay. The sections were examined in a blinded manner under light microscopy (Olympus BX-50; Olympus Optical, Tokyo, Japan). The heart tissues from each group were also embedded in OCT compound (Tissue-Tek Sakura Finetek, Torrance, CA, USA), and the cryosections (4–5 μm) and the cultured HCMs were incubated with the oxidative fluorescent dye dihydroethidine (DHE, 2 μM, Invitrogen). For immunofluorescence analysis, heart sections and cardiomyocytes were incubated with primary antibodies anti-F4/80 (Serotec, Oxford, UK), anti-PEX 5 (peroxisomal biogenesis factor; Invitrogen, Carlsbad, CA), anti-connective tissue growth factor (CTGF), anti-4-hydroxonenal (HNE), anti-KDEL, anti-toll-like receptor (TLR) 4, and anti-perilipin-1/-2 (Abcam, Cambridge, UK)), followed by incubation with fluorescent-dye conjugated secondary antibodies (Alexa Fluor 488-conjugated anti-rabbit IgG (1:1,000, Life Technologies) and Alexa Fluor 555-conjugated anti-rat IgG (1: 1,000, Life Technologies)). For intracellular calcium imaging, Fluo-4 AM, a cell-permeant fluorescent calcium indicator (ThermoFischer Scientific, Waltham, MA, USA) was used. The fluorescent images were examined under a laser scanning confocal microscope system (Carl Zeiss LSM 700, Oberkochen, Germany). Two investigators were blinded to quantify the stained areas of approximately 20 views (200x and 400x magnifications), which were located randomly in the middle portion of the myocardium of each slide (ImageJ software, 1.47 v; NIH).

### Semi quantitative Real-time PCR analysis

Total RNAs from cell lysates or myocardial tissue were isolated. Gene expression was determined by semi quantitative RT-PCR. Primer sequences of *AdipoR1* and *AdipoR2* are as follow: *AdipoR1* forward primer 5′-TTCTTCCTCATGGCTGTGATGT-3′, *AdipoR1* reverse primer 5′-AAGAAGCGCTCAGGAATTCG-3′, and *AdipoR2* forward primer 5′ATAGGGCAGATAGGCTGGTTGA-3′, *AdipoR2* reverse primer 5′GGATCCGGGCAGCATACA-3′.

### Western blot analysis and enzyme activity determination

Mouse heart tissue and cardiomyocytes were extracted with a Pro-Prep Protein Extraction Solution (Intron Biotechnology, Gyeonggi-Do, Korea), following the manufacturer’s instructions. Western assay was performed with specific antibodies for AdipoR1/R2 (Abcam, Cambridge, UK), phospho-Thr^172^ AMPK (pAMPK), total AMPK, phospho-Thr^473^ Akt (pAkt), total Akt, phospho-Ser^1177^ eNOS (p-eNOS), total eNOS, phospho-Ser^428^ liver kinase B1 (pLKB1), total LKB1, phospho-Ser^256^ Forkhead box protein O1 (pFoxO1), total FoxO1 (Cell Signaling Technology, Danvers, MA), arginase I/II, PPARα, TLR4, Perilipin-1/-2, 4-HNE, phosphoinositide 3-kinase (PI3K; Abcam, Cambridge, UK), PPARγ coactivator (PGC)–1α (1:2000; Novus Biologicals, Littleton, CO), CaMKKα/β, sterol regulatory element–binding protein (SREBP)–1c, phosphorylated acetyl-CoA carboxylase (pACC), total ACC, B cell leukemia/lymphoma 2 (BCL-2), BCL-2-associated X protein (BAX), GAPDH (Santa Cruz Biotechnology, Santa Cruz, CA), iNOS (BD Biosciences, San. Jose, CA), PEX 5 (Invitrogen, Carlsbad, CA), and β-actin (Sigma-Aldrich, St. Louis, MO). After incubation with horseradish peroxidase–conjugated antimouse or anti-rabbit IgG (Cell Signaling Technology, Danvers, MA), target proteins were visualized by an enhanced chemiluminescence substrate (ECL Plus; GE Healthcare Bio-Science, Piscataway, NJ).

### Statistical analysis

The data are expressed as means ± SD. Differences between the groups were examined for statistical significance using ANOVA with Bonferroni correction using SPSS version 11.5 (SPSS, Chicago, IL). A *P* value <0.05 was considered statistically significant.

## Results

### AdipoRon improves insulin resistance and cardiac structural and functional parameters without affecting serum adiponectin and glucose levels in *db/db* mice

**Fig. 1:**
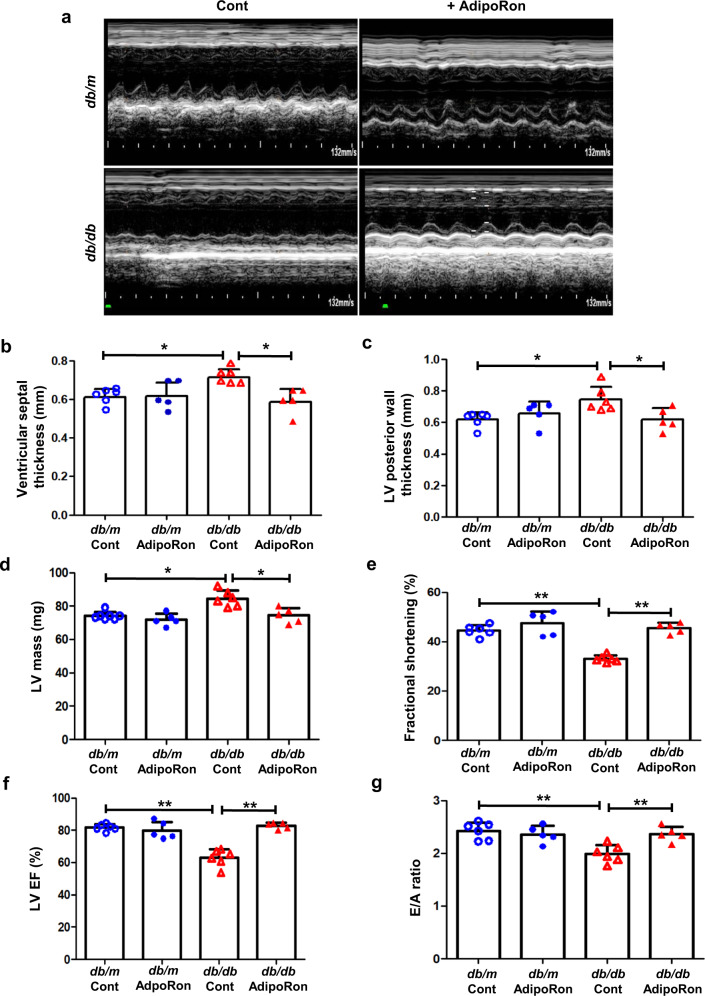
Changes in echocardiographic parameters of diabetic and non-diabetic mice with or without AdipoRon treatment. **a** Representative images of echocardiogram. **b-g** Quantitative analyses of echocardiographic findings according to groups. *P < 0.05, and **P < 0.01 compared with other groups.

Serum adiponectin levels were higher in non-diabetic mice than in diabetic ones. The AdipoRon treatment affected neither plasma glucose, hemoglobin A1c, and adiponectin levels nor serum lipid profiles, including serum total cholesterol, triglycerides, and non-esterified fatty acids, both in non-diabetic and diabetic mice. In contrast, serum insulin level, systemic lipid peroxidation, and oxidative DNA damages as reflected by homeostatic model assessment of insulin resistance and 24-hour-urinary 8-OH-dG concentrations, as well as urinary isoprostane significantly decreased upon AdipoRon treatment. We found no differences in systolic or diastolic blood pressure, and heart rate among the study groups (Table 1). The results of our echocardiographic investigations demonstrated a recovery from left ventricular hypertrophy, left ventricular (LV) posterior wall thickness, and LV mass reduction (Fig. 1a–d). Both systolic and diastolic functional parameters improved; the fractional shortening, LV ejection fraction (EF), and peak E to peak A velocity (E/A) ratio increased in AdipoRon-treated diabetic mice (Fig. 1a, e–g). These results suggest that AdipoRon improves systemic insulin resistance and cardiac hypertrophy and functional parameters without affecting serum adiponectin and glucose levels.

**Table 1:** Biochemical and physical characteristics of diabetic and non-diabetic mice after AdipoRon treatment.

Characteristics	db/m Cont	db/m AdipoRon	db/db Cont	db/db AdipoRon
Body weight (g)	35.4 ± 1.5	36.2 ± 1.7	52.5 ± 5.5***	51.7 ± 3.9***
Heart weight (g)	0.18 ± 0.02	0.18 ± 0.02	0.19 ± 0.03	0.17 ± 0.02*****
FBS (mg/dl)	142 ± 13	140 ± 11	544 ± 82***	539 ± 77***
HbA1c (%)	3.9 ± 0.2	3.8 ± 0.2	11.1 ± 0.5***	10.9 ± 0.7***
Serum insulin (ng/ml)	0.92 ± 0.47	0.96 ± 0.29	10.35 ± 4.39***	1.86 ± 0.73
HOMA_IR_	0.06 ± 0.01	0.06 ± 0.01	2.41 ± 0.05^+^	0.43 ± 0.03*
Total cholesterol (mmol/l)	2.8 ± 0.5	2.6 ± 0.4	3.7 ± 0.6*	3.4 ± 0.4*
Triacylglycerols (mmol/l)	1.32 ± 0.11	1.22 ± 0.21	2.38 ± 0.32***	1.72 ± 0.33*
Non-esterified fatty acid (mmol/l)	0.67 ± 0.22	0.67 ± 0.19	1.42 ± 0.20*	1.21 ± 0.15*
Systolic BP (mmHg)	120 ± 8	117 ± 12	117 ± 16	109 ± 10
Diastolic BP (mmHg)	83 ± 20	75 ± 15	66 ± 20	65 ± 10
Heart rate (/min)	537 ± 45	440 ± 70****	478 ± 31	457 ± 25
Serum adiponectin (μg/ml)	11.3 ± 1.1	10.9 ± 1.2	4.3 ± 0.4***	4.4 ± 0.7***
Urinary 8-OH-dG (ng/24 hr)	39.3 ± 15.5	38.8 ± 17.9	205.4 ± 66.9***	65.4 ± 25.2
Urinary isoprostane (ng/24 hr)	4.8 ± 1.7	4.6 ± 2.1	46.7 ± 18.6^†^	21.5 ± 9.8*

All values are depicted as mean ± SD.

*Cont* Control, *FBS* Fasting blood sugar, *HbA1c* Hemoglobin A1c, *HOMAIR* Homeostatic model assessment of insulin resistance, *BP* Blood pressure, *8-OH-dg* 8-hydroxy-deoxyguanosine.

Statistical analysis was done using Two-Way-ANOVA with Bonferroni post-test. *P < 0.05, **P < 0.01, and ***P < 0.001 compared with other groups. ****P < 0.05 compared with *db/m* Cont, *****P < 0.05 *compared with db/db* Cont.

### AdipoRon improves diabetes-induced cardiac damage; it reduces intracardiac fibrosis, inflammation, and apoptosis in *db/db* mice

**Fig. 2:**
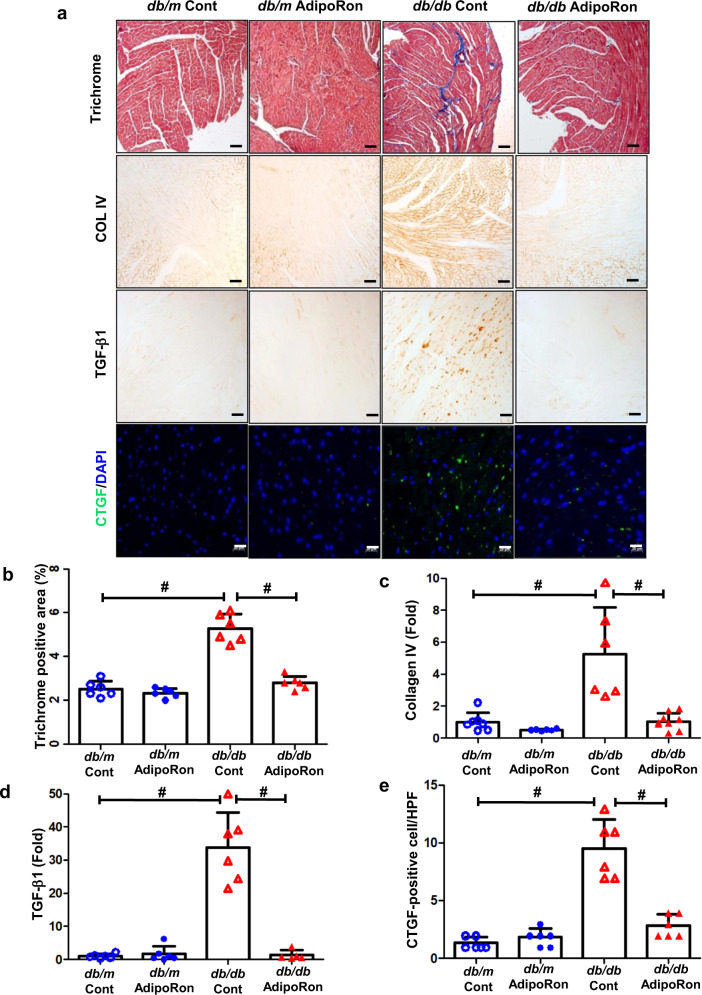

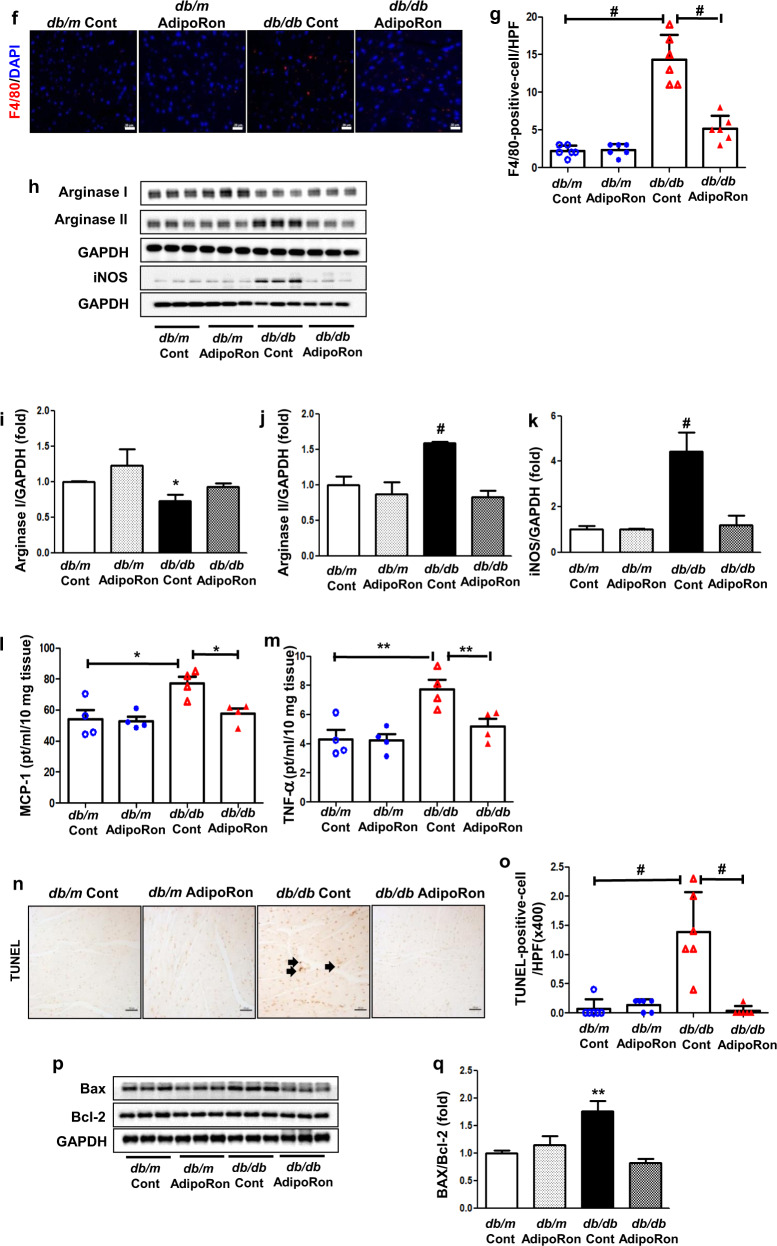
Changes in the myocardiac phenotypes; fibrosis, inflammation, and apoptosis of diabetic and non-diabetic mice with or without AdipoRon treatment. **a** Representative sections stained with Masson trichrome to estimate the trichrome positive area (%), immunohistochemical staining for type IV collagen, TGF-β1-positive area, and immunofluorescence staining of CTGF-positive cells. **b**–**e** Quantitative analyses of the representative sections according to groups. **f** Representative images of immunofluorescence staining of F4/80-positive cells. **g** Quantitative analyses of F4/80-positive cells. **h** Representative images of western blotting of arginase I, arginase II, iNOS, and GAPDH levels. **i**–**k** Quantitative analyses of representative western blotting images according to groups. **l**, **m** Quantitative analyses of MCP-1 and TNF-α in the cardiac tissues according to groups. **n** Immunohistochemical staining of TUNEL-positive cells. **o** Quantitative analyses of immunohistochemical staining according to groups. **p** Representative images of western blotting of Bax/Bcl-2. **q** Quantitative analyses of Bax/Bcl-2 according to groups *P < 0.05, **P < 0.01, and ^#^*P* < 0.001 compared with other groups. Scale bars, 20 μm (in **a**, **f**)/ 100 μm (in **n**).

AdipoRon treatment improved cardiac fibrosis; the increased expression levels of Col IV and TGF-β1, and the increased extent of the trichrome-positive and CTGF-positive area, which play crucial role in the development of heart fibrosis, decreased in the AdipoRon-treated diabetic mice group (Fig. 2a–e). Inflammation was alleviated by the AdipoRon treatment; the increased MCP-1, TNF-α, and F4/80- positive cell infiltration in the myocardium decreased, with corresponding decreases in arginase II and iNOS levels and increase in arginase I, suggesting that type 1 macrophage (M1) is responsible for the inflammatory reaction in the cardiac tissue of diabetic mice (Fig. 2f–m). We could observe an improvement in intracellular apoptosis following the AdipoRon treatment, displayed by the reduced level of TUNEL-positive cell infiltration and Bax/Bcl-2 expression in the cardiac tissues of diabetic mice (Fig. 2n–q).

### AdipoRon enhances ceramide metabolism and reduces oxidative stress in association with decreased intracardiac perilipin and TLR4 expressions in *db/db* mice

**Fig. 3:**
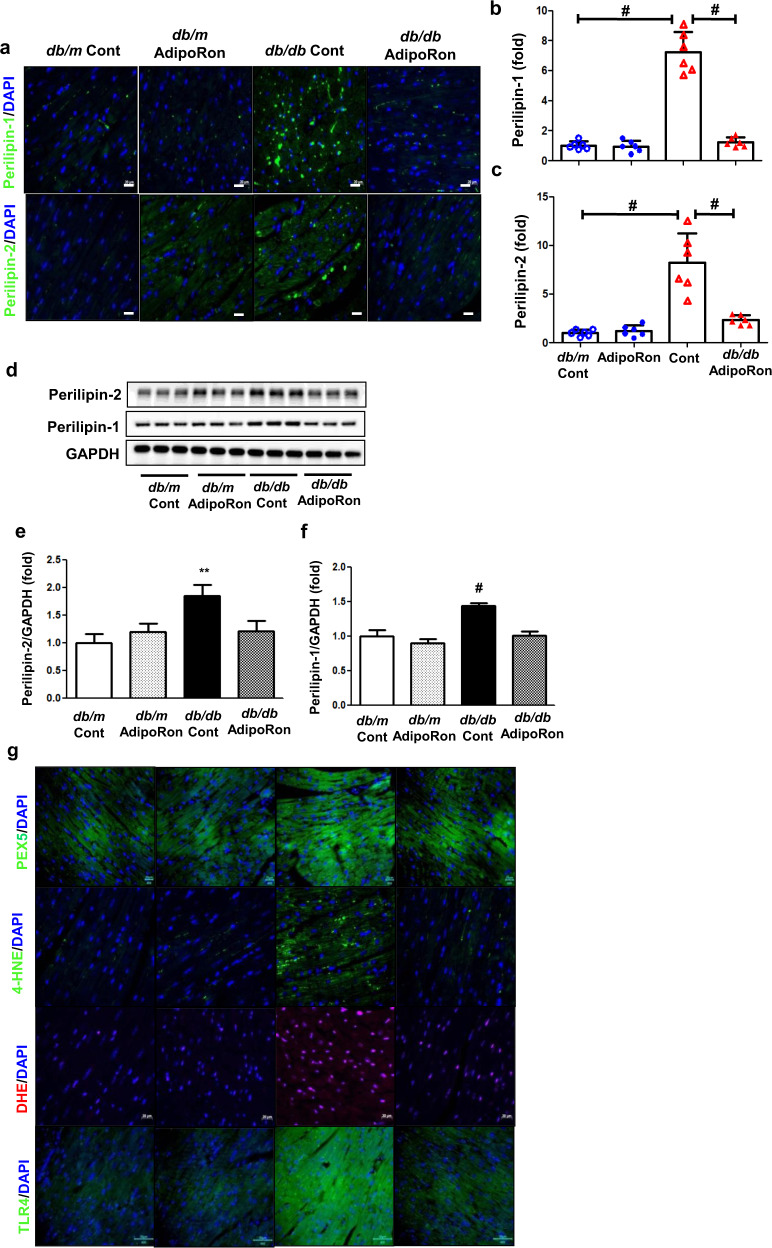

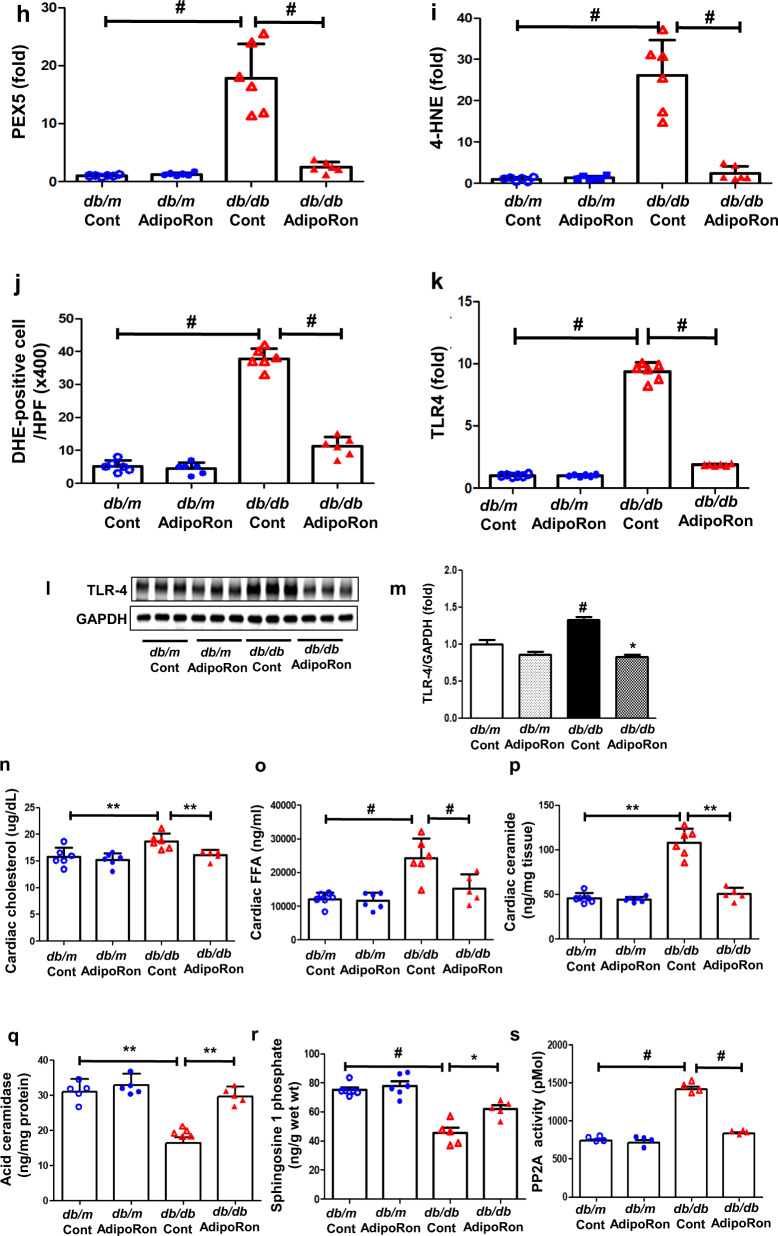
Changes in ceramide metabolism and its associated pathways in diabetic and non-diabetic mice with or without AdipoRon treatment. **a** Representative images of immunofluorescence staining of perilipin-1/-2. **b, c** Quantitative analyses of perilipin-1/-2 according to groups. **d** Representative images of western blotting of perilipin-1/-2, and GAPDH levels. **e**, **f** Quantitative analyses western blotting images according to groups. **g** Representative images of immunofluorescence staining of PEX5, 4-HNE, DHE-positive cells, and TLR4 level (**h**–**k**) Quantitative analyses of immunofluorescence images according to groups. **l** Representative images of western blotting of TLR4 and GAPDH levels. **m** Quantitative analyses of TLR-4 expression according to groups. **n**–**s** Quantitative analyses of cholesterol, FFA, ceramide, acid ceramidase, sphingosine 1 phosphate, and PP2A activity in the cardiac tissues. *P < 0.05, **P < 0.01, and ^#^*P* < 0.001 compared with other groups. Scale bars, 20 μm for perilipin-1/-2 (in **a**) and 50 μm for TLR4 (in g).

A marked accumulation of intracardiac lipid droplets, demonstrated by the perilipin-1 and 2 expressions in diabetic mice decreased following the AdipoRon treatment (Fig. 3a–f). AdipoRon treatment reduced the increased expression of intracardiac TLR4 which stimulates ceramide biosynthesis via IKKβ in diabetic mice. It was accompanied by significant reductions in the increased intracardiac peroxisome biogenesis, oxidative stress and lipid peroxidation in AdipoRon-treated diabetic mice, demonstrated by the PEX5-positive cells, DHE-positive cells, and 4-HNE expression (a stable and reactive lipid peroxidation product), respectively (Fig. 3g–m). Finally, ceramide metabolism was enhanced; the increased ceramidase activity hydrolyzed ceramide to form sphingosine, leading to an increase in the S1P: ceramide ratio, while decreasing PP2A activity (Fig. 3n–s). We also analyzed further changes in the intracardiac ceramide profiles. The quantitative analysis of the ceramide subtypes revealed that fractions of the increased sphingosine (CX-NS)- and dihydrosphingosine (CX-NDS)-conjugated non-hydroxy fatty acid, C16-, C18-, C20-, C24-NS/NDS decreased in AdipoRon-treated diabetic mice (Supplementary Figs. 1a–f).

### AdipoRon modulates intracardiac AdipoR1/AdipoR2 expressions via the pAMPK-FoxO1 dependent pathway in *db/db* mice; activation of associated primary signaling pathways enhances downstream target molecules involved in lipid metabolism, mitochondrial biogenesis, and endothelial dysfunction

**Fig. 4:**
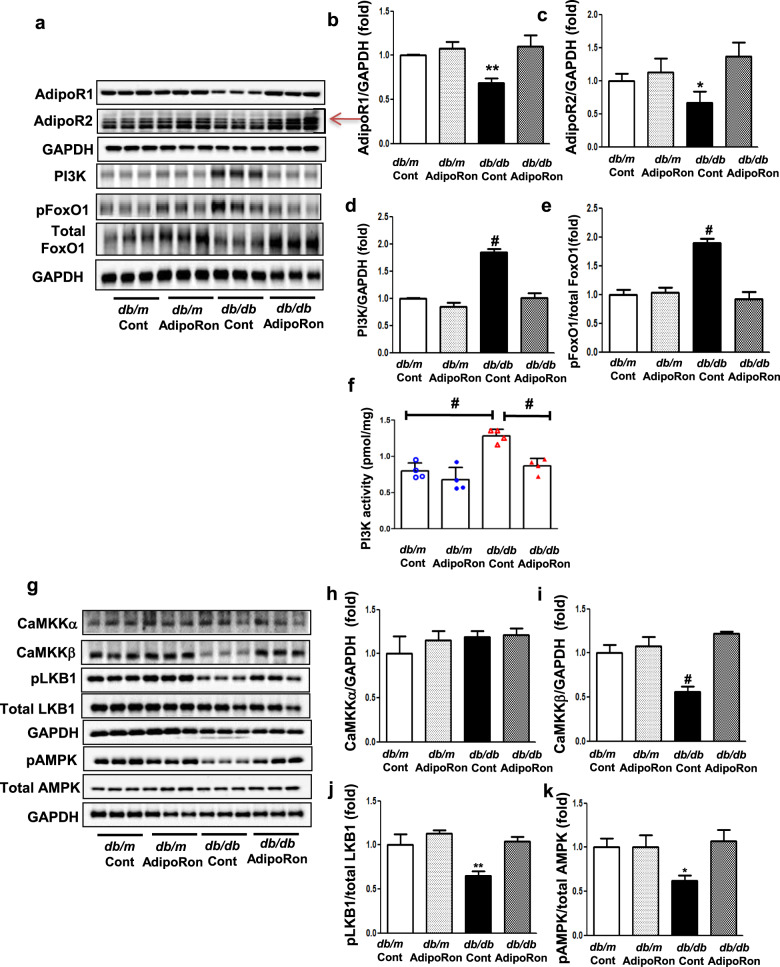

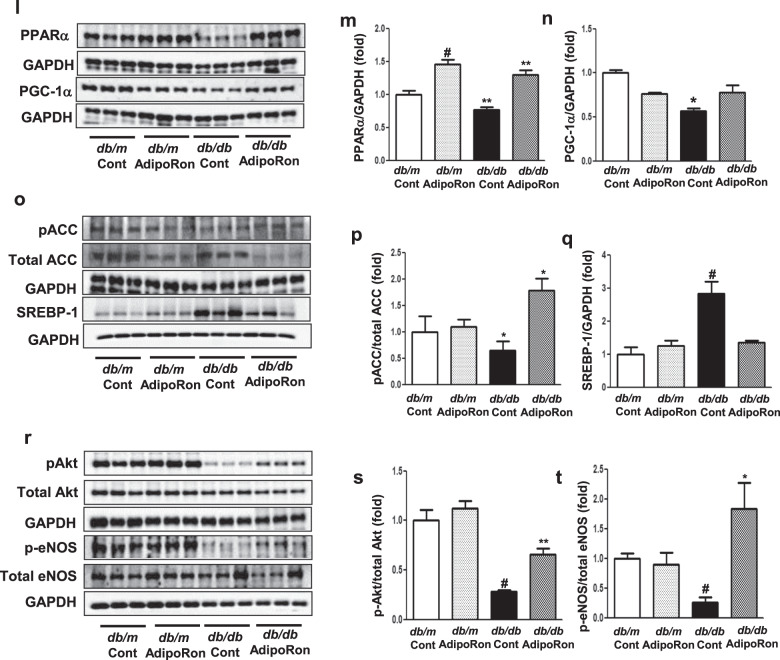
Changes in the expression of adiponectin receptors and their associated downstream signaling pathways in diabetic and non-diabetic mice with or without AdipoRon treatment. **a** Representative images of western blotting of AdipoR1, AdipoR2, PI3K, pFoxO1, total FoxO1, and GAPDH levels. **b**–**e** Quantitative analyses of western blotting images according to groups. **f** Quantitative analyses of PI3K activity according to groups. **g** Representative images of western blotting of CaMKKα, CaMKKβ, phosphorylated Ser^431^LKB1, total LKB1, phosphorylated AMPK Thr^172^, total AMPK and GAPDH levels. **h**–**k** Quantitative analyses of western blotting images according to groups. **l** Representative images of western blotting of PPARα, PGC-1α, and GAPDH levels. **m**, **n** Quantitative analyses according to groups. **o** Representative images of western blotting of phosphorylated ACC, total ACC, SREBP-1c, and GAPDH levels. **p**, **q** Quantitative analyses according to groups. **r** Representative images of western blotting of phosphorylated Akt, total Akt, phosphorylated Ser^1177^eNOS, total eNOS, and GAPDH levels. **s**, **t** Quantitative analyses according to groups. *P < 0.05, **P < 0.01, and ^#^*P* < 0.001 compared with other groups.

AdipoRon treatment restored the diabetes-induced decreases in intracardiac AdipoR1 and AdipoR2 expressions to levels comparable to those in control *db/m* mice. These were accompanied by decreases in PI3K activity and pFoxO1/total FoxO1 level, suggesting its potential mechanistic role in modulating AdipoR expressions (Fig. 4a–f). AdipoRon treatment increased the expressions of CaMKKβ and phosphorylated LKB1 but not that of CaMKKα, and activated phosphorylated AMPK and PPARα, which are the primary downstream targets of AdipoR1 and AdipoR2, respectively (Fig. 4g–m). Downstream pathways associated with activated PGC-1α increased the phosphorylation of ACC and reduced the expression of SREBP-1c, which is expected to exert pro-metabolic effects by enhancing fatty acid oxidation and mitochondrial biogenesis in the cardiac tissue (Fig. 4l–q). AdipoRon treatment further promoted Akt phosphorylation, leading to enhanced NO bioavailability (Fig. 4r–t).

## IN VITRO STUDIES

### AdipoRon activates the phosphorylated Ser^431^LKB1/phosphorylated Thr^172^AMPK/PPARα and their downstream targets through AdipoR agonism in HCMs

**Fig. 5:**
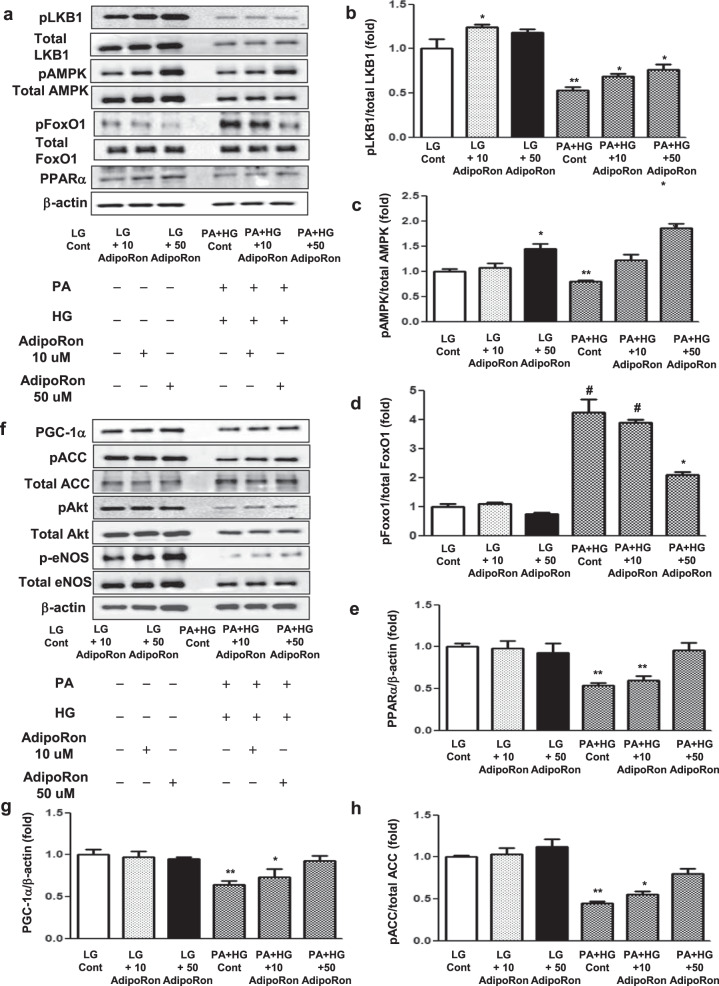

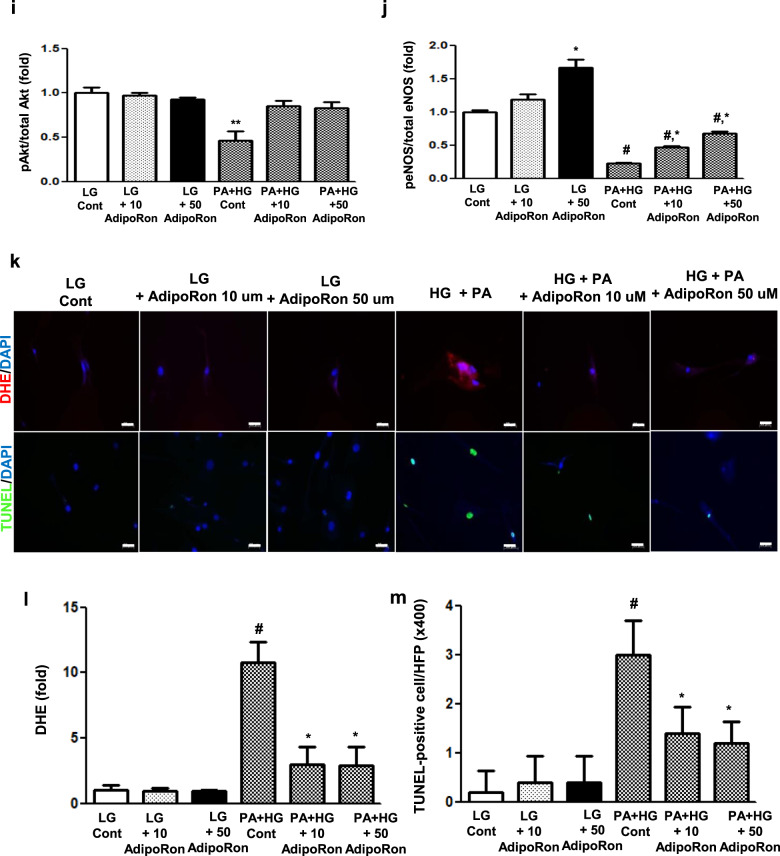
Effect of AdipoRon on downstream signaling pathways in human cardiomyocytes cultured in low- or high-glucose (LG, HG) and palmitate (PA) media with or without AdipoRon. **a** Representative images of western blotting of pLKB1, total LKB1, pAMPK, total AMPK, pFoxO1, total FoxO1, PPARα, and β-actin levels**. b**–**e** Quantitative analyses according to groups. **f** Representative images of western blotting of PGC-1α, phosphorylated ACC, total ACC, pAkt, total Akt, phosphorylated Ser^1177^eNOS, total eNOS, and β-actin levels. **g**–**j** Quantitative analyses according to groups. **k** Representative images of immunofluorescence staining of DHE expression and TUNEL-positive cells. **l**, **m** Quantitative analyses according to groups. *P < 0.05, **P < 0.01, and ^#^*P* < 0.001 compared with other groups. Scale bars, 20 μm (in k).

To determine whether these results were direct effects exerted on cardiac myocytes or might be due to systemic changes, we performed a series of in vitro experiments on HCMs. HCMs grown in both HG and PA media, an environment mimicking conditions similar to type 2 diabetes, were treated with AdipoRon in a dose-dependent manner. AdipoRon decreased pFoxO1/total FoxO1 level and activated pLKB1, pAMPK, and PPARα pathways, enhancing the expression of their key downstream target molecules such as PGC-1α, p-ACC, p-eNOS, and p-Akt in a dose-dependent manner, which further improved oxidative stress and cellular apoptosis, shown by the decreased expressions of DHE and TUNEL in HCMs (Fig. 5a–m).

### AdipoRon enhances ceramide metabolism and reduces oxidative stress in association with decreased perilipin and TLR4 expressions in HCMs

**Fig. 6:**
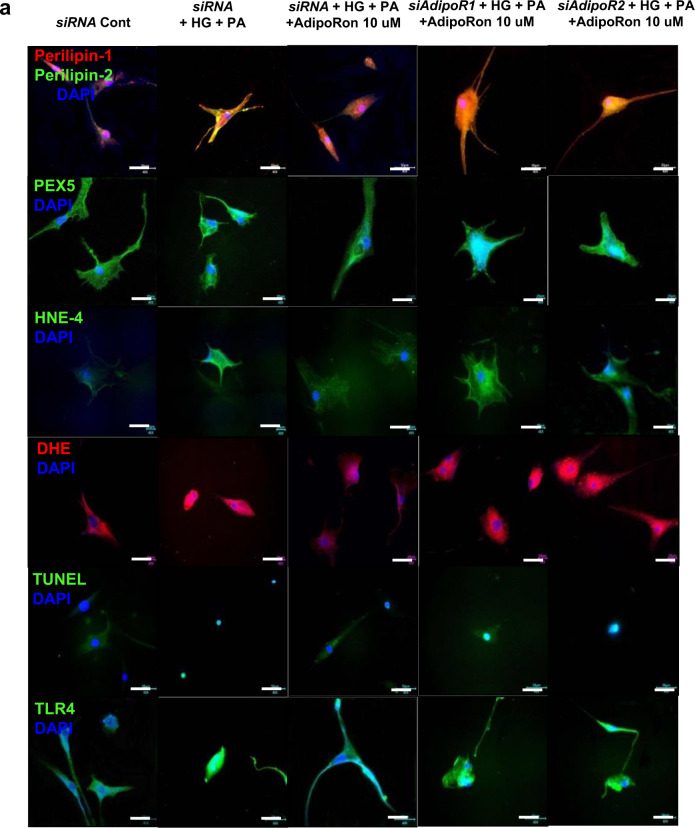

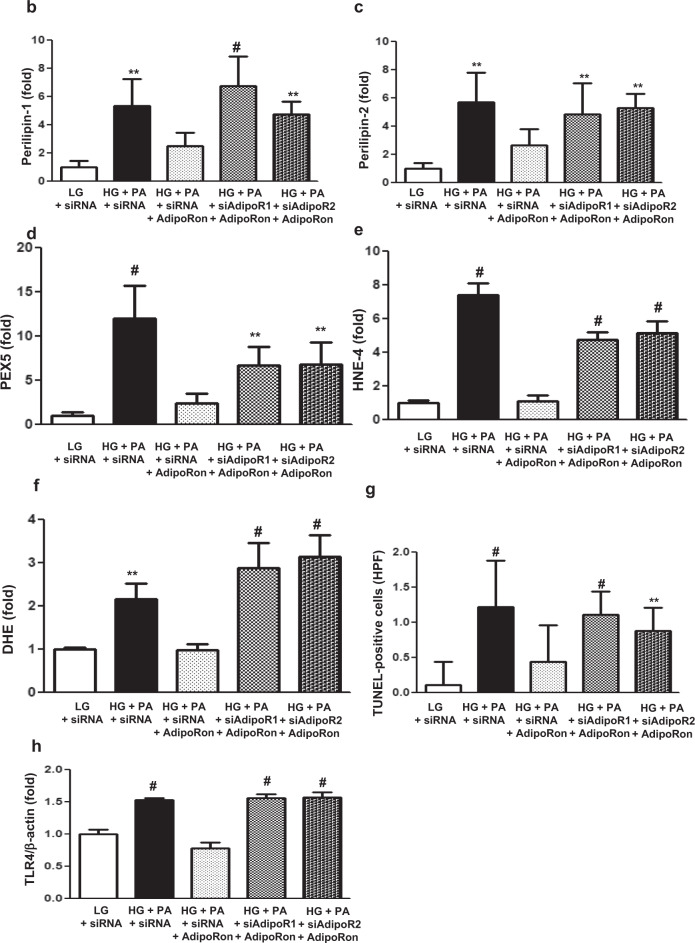

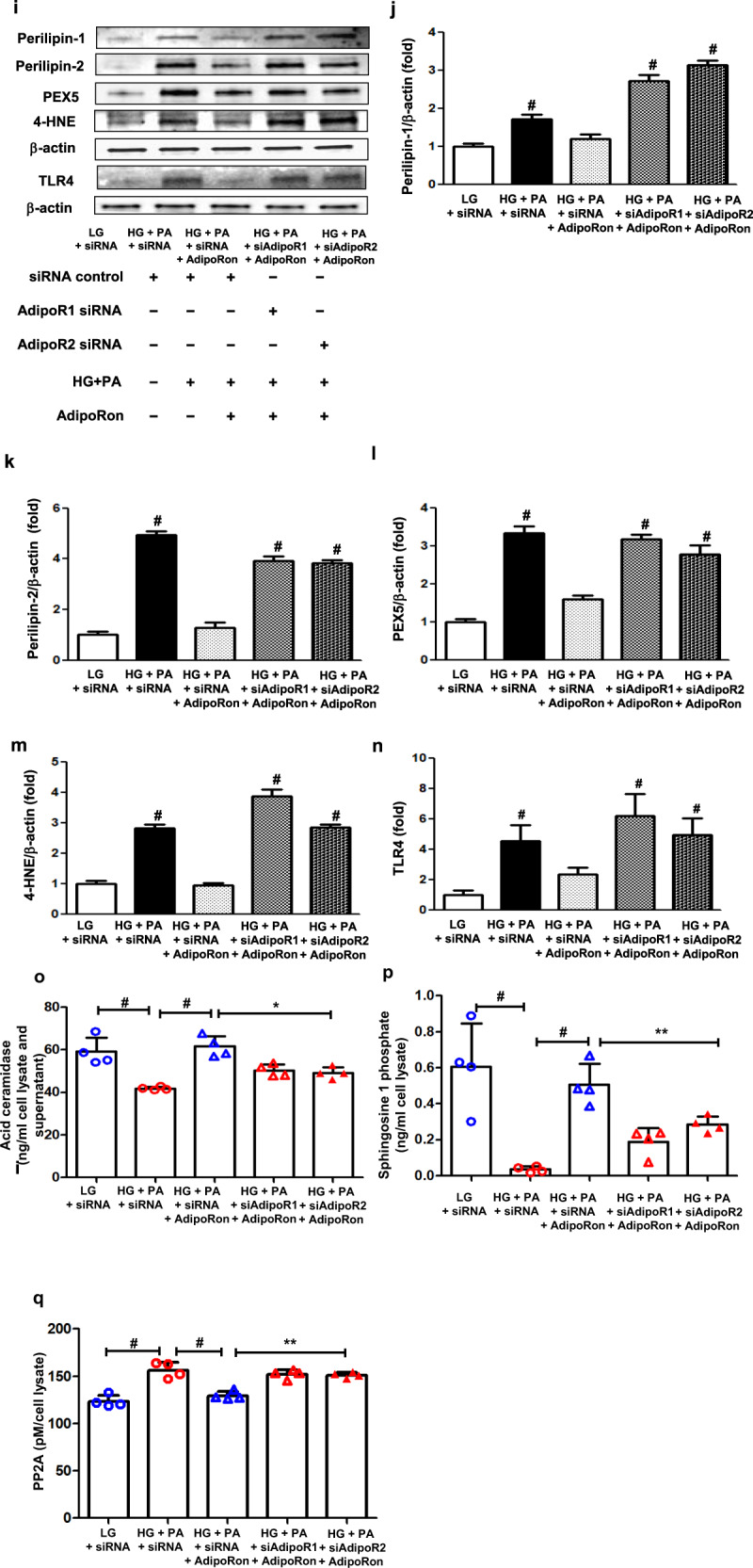
Effect of AdipoR1 and AdipoR2 siRNAs on oxidative stress and ceramide metabolism in human cardiomyocytes cultured in low- or high-glucose and palmitate media with or without AdipoRon. **a** Representative images of immunofluorescence staining of perilipin-1/-2, PEX5, HNE4, DHE, TUNEL, and TLR4 expressions. **b**–**h** Quantitative analyses according to groups. **i** Representative images of western blotting of perilipin-1/-2, PEX5, HNE4, TLR4, and actin levels. **j**–**n** Quantitative analyses according to groups. **o**–**q** Quantitative analyses of acid ceramidase, sphingosine 1 phosphate, and PP2A activity according to groups. *P < 0.05, **P < 0.01, and ^#^*P* < 0.001 compared with other groups. Scale bars, 50 μm (in **a**).

To determine which AdipoR was responsible for activating the downstream effectors of the pathway, HCMs cultured in the HG and PA media, were transfected with small interfering RNAs (siRNAs), thereby silencing the genes encoding *AdipoR1* and *AdipoR2*. The transfection of the myocytes with *AdipoR1* siRNA suppressed AdipoR1 expression and with *AdipoR2* siRNA suppressed AdipoR2 expression by about 50%, respectively (Supplementary Figs. 2a–c). The immunoblotting and immunofluorescence studies of HCMs revealed that the increased expression of perilipin-1/-2, PEX5, HNE-4, DHE, and TUNEL did not decrease in cells transfected with both *AdipoR1* and *AdipoR2* siRNAs, confirming that the expressions of these downstream target molecules are AdipoR-dependent (Fig. 6a–n). The AdipoRon treatment did not decrease TLR4 expression in cells transfected both with *AdipoR1* and *AdipoR2* siRNAs (Fig. 6a, h, I, and n). The acid ceramidase and S1P expressions did not increase and that of PP2A did not decrease with AdipoRon treatment in HCMs transfected with both *AdipoR1* and *AdipoR2* siRNAs (Fig. 6o–q).

### AdipoRon modulates AdipoR1/AdipoR2 expressions via the pAMPK-FoxO1-dependent pathway in HCMs; activation of associated primary signaling pathways enhances downstream target molecules involved in lipid metabolism, mitochondrial biogenesis, and endothelial dysfunction

**Fig. 7:**
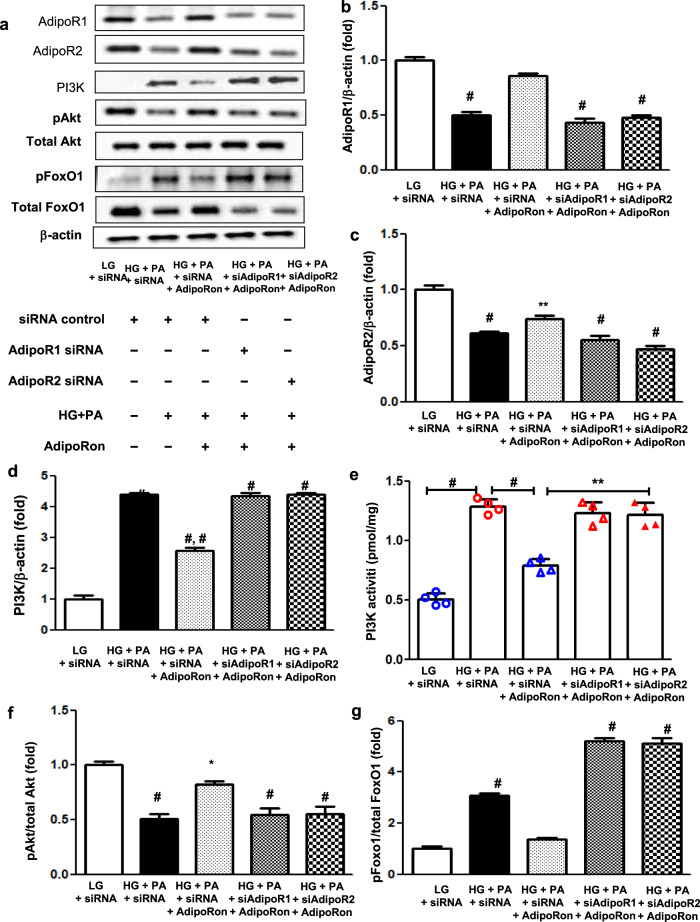

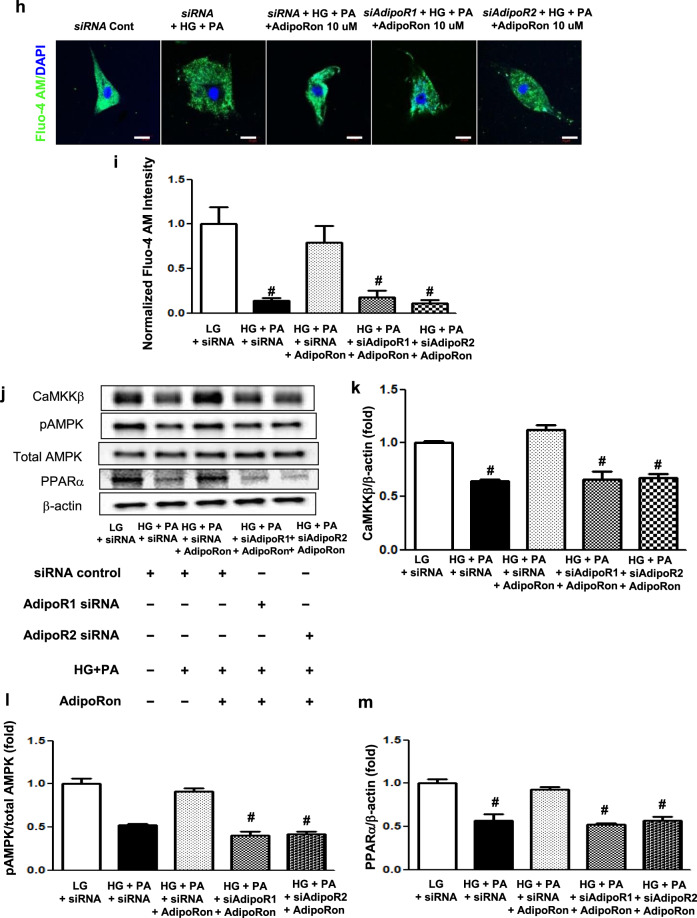
Effect of AdipoR1 and AdipoR2 siRNAs on PI3K/FoxO1 and associated primary signaling pathways in human cardiomyocytes cultured in low- or high-glucose and palmitate media with or without AdipoRon. **a** Representative images of western blotting of AdipoR1, AdipoR2, PI3K, pAkt, total Akt, pFoxO1, total FoxO1, and β-actin levels. **b**–**g** Quantitative analyses according to groups. **h** Representative images of immunofluorescence staining of Fluo-4 AM. **i** Quantitative analyses if Fluo-4 AM according to groups. **j** Representative images of western blotting of CaMKKβ, pAMPK, total AMPK, PPARα, and β-actin levels. **k**–**m** Quantitative analyses according to groups. *P < 0.05, **P < 0.01, and ^#^*P* < 0.001 compared with other groups. Scale bars, 20 μm (in **h**).

AdipoRon treatment did not decrease PI3K activity and pFoxO1 levels or increase each AdipoR level in cells transfected both with *AdipoR1* and *AdipoR2* siRNAs in HG and PA media (Fig. 7a–g). Nor did it increase Fluo-4 AM, CaMKKβ, pAMPK and PPARα expression levels in cardiomyocytes transfected either with *AdipoR1* or *AdipoR2* siRNA, confirming that the expressions of these downstream target molecules are AdipoR-dependent (Fig. 7h–m).

## Discussion

**Fig. 8:**
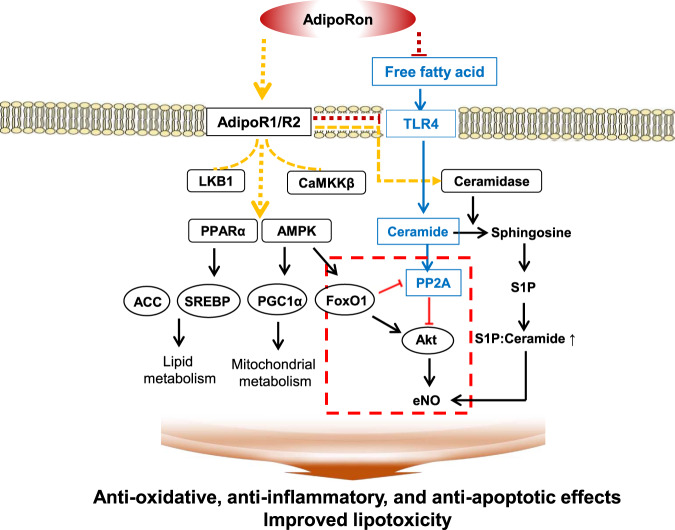
Cardiomyocyte survival and proliferation by adiponectin receptor agonism. Adiponectin receptor agonism activates CaMKKβ, LKB1, AMPK and PPARα. It also has ceramidase activity and can catalyze the conversion of ceramide to sphingosine, which produces S1P, subsequently increasing S1P to ceramide ratio that further ameliorates endothelial dysfunction through increased NO level. Activation of associated downstream pathways exert prometabolic effects by enhancing fatty acid oxidation and mitochondrial biogenesis. Red dottedlines: Constitutive activation of FoxO1 by AMPK represses PP2A and its interaction with Akt, subsequently increasing Akt phosphorylation, which would otherwise have been dephosphorylated by continuous inhibitory effect of PP2A with subsequent inhibition of eNOS through ceramide biosynthesis. The net result is the improvement of ceramide-induced oxidative stress via pro-metabolic effects of adiponectin agonism in which pAMPK-induced FoxO1 activation might play a crucial role in modulating its receptor expressions.

In the current study, we demonstrated that AdipoR agonism by AdipoRon decreased systemic insulin resistance and increased cardiac acid ceramidase activity that rendered both phenotypic and functional improvements in the myocardium of diabetic mice. AdipoRon increased AdipoR1 and AdipoR2 expressions, acid ceramidase activity, and associated signaling pathways involving pAMPK-PPARα. Downstream target molecule expressions including PGC-1α-pACC increased, and that of S1P increased in association with decreased PP2A activity and phosphorylation of Akt and eNOS increased, which were subsequently followed by a reduced degree of ceramide-induced lipotoxicity, oxidative stress, inflammation, and apoptotic cell deaths (Fig. 8).

Our analysis suggests that AdipoRs might possess their innate activity to induce ceramidase upon activation, which serves as a regulator of ceramide physiology and S1P balance. Ceramide acts upstream of S1P by inhibiting Akt phosphorylation: it dephosphorylates Akt via PP2A, which impairs NO bioavailability, leading to reduced cell viability and endothelial dysfunction [19]. In contrast, S1P can upregulate Bcl-2 family members while downregulating Bax, which negatively mediates apoptosis [20, 21]. AdipoRon treatment increased the phosphorylation of Akt by reducing PP2A levels, which decreased the phosphorylation of Bcl-2, and at the same time, the catalytic subunit of PP2A inhibited Bcl-2, leading to an overall suppression of the Bcl-2/Bax ratio in diabetic hearts, coupled with the improvement of intrinsic apoptosis in the relevant tissue. In the current study, the reduced ceramide contents in the target organ by AdipoRon, along with the increased S1P levels, could promote cell survival via increased Akt phosphorylation.

The downregulation of ceramide also modulates and improves mitochondrial function. Ceramide could disrupt mitochondrial membrane structure by reducing state 4 respiration and inhibiting electron transport complex I, which elicits elevated reactive oxygen species (ROS) levels and increases membrane permeability, further inducing apoptotic pathways [22]. Ceramides are also thought to directly inhibit complex III, thereby generating ROS and inflammation [6]. Meanwhile, S1P can enhance the stability of mitochondrial membranes by blocking the translocation of Bax to the mitochondria [23]. The current study demonstrated evidences for a net reduction in oxidative stress, lipid peroxidation levels which might in part be a consequence of enhanced mitochondrial oxidative capacity through ceramide reduction.

Confirming that ceramide metabolites promote a broad spectrum of deleterious consequences, further issues should be addressed, such as which subspecies are most responsible for disease onset and what is the most quantitatively significant role they play. Our results are in good agreement with previous data [24], showing that the AdipoRon treatment ameliorated ceramide-induced toxicity through decreases in the contents of total and whole fractions of ceramides (from C16 to C28), indicating that targeting long acyl chain length ceramides via acid ceramidase might serve as a potential selective therapeutic option.

Apart from various prometabolic effects conferred by ceramidase activation, AdipoR1 and AdipoR2 upregulate their primary downstream signaling pathways, including pAMPK-PPARα-PGC1α. Changes in the associated downstream target molecules resulted in the improvement of lipid metabolism; moreover, increased phosphorylation of ACC and decreased expression of SREBP-1c altered the expression of perilipin at both cellular and tissue levels. Recently, adiponectin and AdipoR1 have shown to induce extracellular Ca^++^ influx that leads to an increased PGC-1α activity and enhanced mitochondrial biogenesis [12, 13]. Our study demonstrates that adiponectin receptor agonism promoted the deacylation of ceramide and resultant sphingosine and S1P increased intracellular Ca^++^ and activated AMPK via stimulation of CaMKKβ. This is in line with the previous literature, consistently emphasizing that the ceramidase activity is upstream of pAMPK-PPARα.

An in vitro study using *AdipoR1* and *AdipoR2* siRNAs in HCMs highlighted the same significant role-play assigned to both AdipoR1 and AdipoR2 in exerting cardioprotective effects under the presence of excess HG and PA media, which mimics the condition similar to type 2 diabetes. Expression of further downstream effectors involved in ceramide metabolism including S1P and PP2A did not increase and decrease in HCMs transfected with *AdipoR1* or *AdipoR2* siRNA even after AdipoRon treatment, respectively, suggesting that dual activation of AdipoR1/AdipoR2 is required for exerting prometabolic effects. Moreover, sustained increase in the level of TLR4 expression, which initiates an inflammatory cascade essential to ceramide biosynthesis and metabolic impingement, even in cardiomyocytes transfected with both *AdipoR1* and *AdipoR2* siRNAs denotes a functioning inflammatory response necessary to drive ceramide metabolism [25]. Indeed, recent works by Holland et al. have identified ceramide as a critical factor for TLR4-mediated antagonism of insulin action and the anti-inflammatory effects of adiponectin may therefore be directly associated with ceramide depletion [26].

We then sought for a mechanism by which AdipoRon enhanced the expression of intracardiac AdipoRs. Based on the previous literature, insulin inhibits myocardial AdipoR1 expression via PI3K/Akt/FoxO1 pathway and FoxO1 mediates AdipoR1 transcription by binding to its promoter directly [27–29]. Our study showed reduced expression of pAkt despite of high serum insulin levels and intracardiac PI3K activity in *db/db* control mice (Fig. 4s), suggesting presence of a significant insulin resistance. In contrast, current in vitro study with HG and PA media showed reduced AdipoR expression which was reversed by AdipoRon treatment, suggesting that there might be insulin independent mechanisms involved in the regulation of AdipoR expression as well. Recent studies showed that the cardioprotective effects of AMPK activation against cardiac hypertrophy are mediated by the FoxO1/MuRF1 pathway [30]. Moreover, continuous activation of FoxO1 enhances Akt activity, and inhibits insulin signaling and cell growth, which prevents hypertrophic cardiac growth [31]. FoxO1 is generally known as a downstream target of insulin, however, its role as an upstream signaling regulator governing insulin sensitivity and glucose metabolism in the heart has been elucidated. It is clearly demonstrated that changes in FoxO activity have a repressive effect on insulin signaling in cardiomyocytes through inhibition of protein phosphatases, which led to Akt activation, reduced insulin resistance and impaired glucose metabolism [32]. In line with these, our current study suggests that agonistic effect of AdipoRon on its receptors may have rendered a constitutive activation of FoxO1 by pAMPK, which led to repress PP2A and its interaction with Akt, subsequently increasing Akt phosphorylation, resulting in the improvement of ceramide-induced oxidative stress via increased expression of AdipoR1/R2 in the heart and in cardiomyocytes. In this context, we reason that pAMPK-induced FoxO1 activation might play a crucial role in modulating AdipoRs expression.

In conclusion, our results demonstrated that disturbed adiponectin–sphingolipid signaling is implicated in the etiology of the lipotoxic environment of diabetic cardiomyopathy in relation to oxidative stress, inflammation, and apoptotic cell death. Apart from the pro-metabolic role play assigned to AdipoR agonism in improving systemic insulin resistance and oxidative stress, we would like to underscore its target-organ specific effect via activating AdipoR1 and AdipoR2 expressions in the myocardium, independent of the systemic effects of adiponectin. AdipoRon, an orally active and synthetic AdipoR agonist increased expression of AdipoR1 and AdipoR2 through pAMPK-FoxO1-Akt-PP2A signaling, and increased acid ceramidase activity-mediated cellular S1P-ceramide rheostat, a key regulator of cellular health, as well as activated their primary downstream signaling pathway involving pAMPK-PPARα/PGC-1α in the heart. These findings suggest adiponectin receptor-mediated ceramidase activity as a primary signaling mechanism by which AdipoRon elicits its broad spectrum of prometabolic effects in the heart.

## Supplementary information

Supplementary Figure Legends

Supplementary Figure S1

Supplementary Figure S2

Supplementary Figure S3

Supplementary Figure S4

Supplementary Figure S5

Supplementary Figure S6

Supplementary Figure S7

Supplementary Figure S8

Supplementary Figure S8

Checklist

## Data Availability

All data and materials used for this study are displayed or could be displayed upon request.
