# Supplementary material for: Adiponectin receptor agonist ameliorates cardiac lipotoxicity via enhancing ceramide metabolism in type 2 diabetic mice
Source: Cell Death Dis. 2022 Mar 30;13(3):282. doi: 10.1038/s41419-022-04726-8 (PMC8964809; doi:10.1038/s41419-022-04726-8)
Supplement: Supplementary file 1 — Supplementary Figure Legends [file 41419_2022_4726_MOESM1_ESM.docx]

**Supplementary Figure S1.**

**a-f** Measured value set of ceramide subtypes and by-products incurred by ceramide metabolism. *^*^P* < 0.05, *^**^P* < 0.01, and ^#^*P* < 0.001 compared with other groups.

**Supplementary Figure S2.**

**a-c** Representative images of real time PCR, western blotting and quantitative analyses of siRNA control and siRNA *AdipoR1/AdipoR2*. ^#^*P* < 0.001 compared with other groups.

**Supplementary Figure S3.**

Full-length uncropped original Western blots in Fig. 2h and 2p

**Supplementary Figure S4.**

Full-length uncropped original Western blots in Fig. 3d and 3l

**Supplementary Figure S5.**

Full-length uncropped original Western blots in Fig. 4a, 4g, 4l, 4o, and 4r

**Supplementary Figure S6.**

Full-length uncropped original Western blots in Fig. 5a and 5f

**Supplementary Figure S7.**

Full-length uncropped original Western blots in Fig. 6i

**Supplementary Figure S8.**

Full-length uncropped original Western blots in Fig. 7a and 7i

**Supplementary Figure S9.**

Full-length uncropped original Western blots in Supplementary Fig. S2
