# Supplementary figures and images for: Adiponectin receptor agonist ameliorates cardiac lipotoxicity via enhancing ceramide metabolism in type 2 diabetic mice
Source: Cell Death Dis. 2022 Mar 30;13(3):282. doi: 10.1038/s41419-022-04726-8 (PMC8964809; doi:10.1038/s41419-022-04726-8)

**a**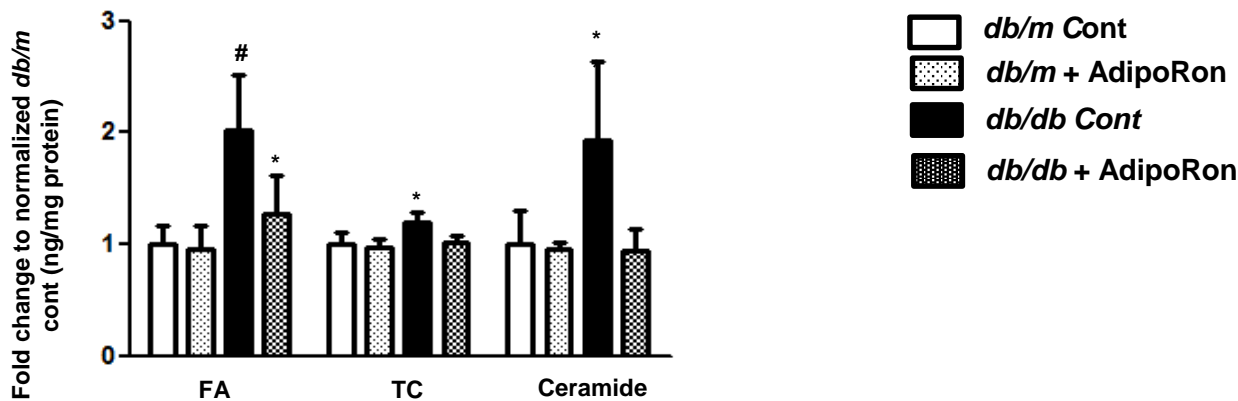**b**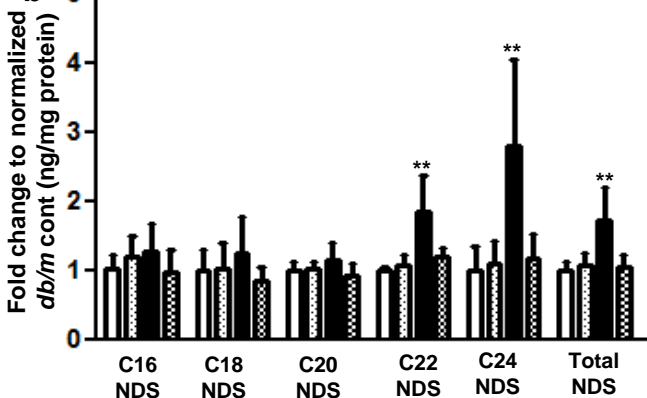**c**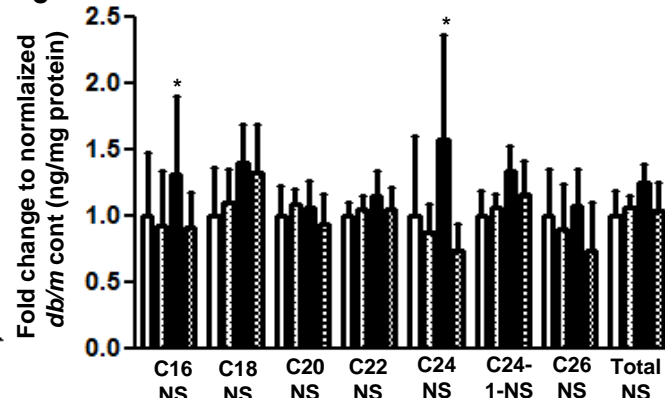**d**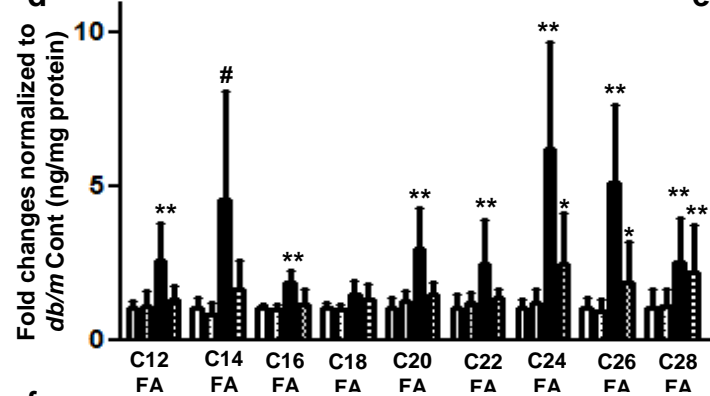**e**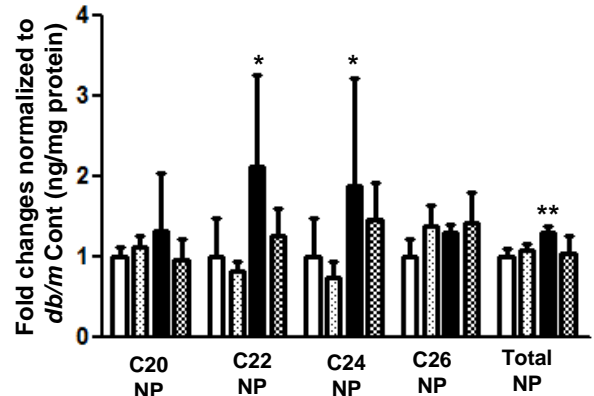**f**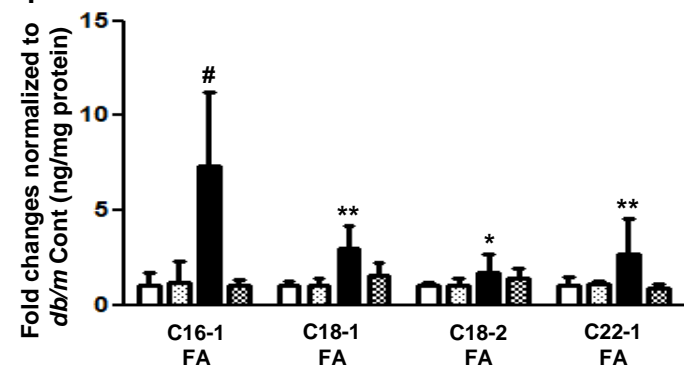

Supplement: Supplementary file 2 — Supplementary Figure S1 [file 41419_2022_4726_MOESM2_ESM.pdf]

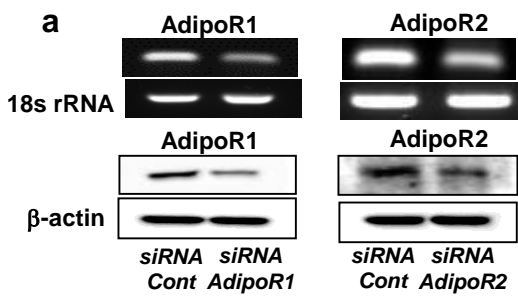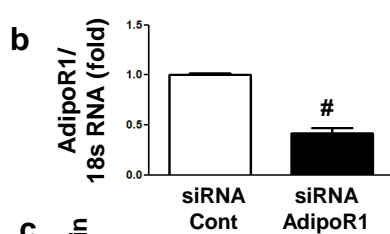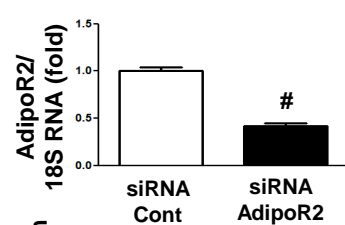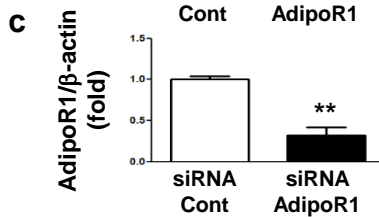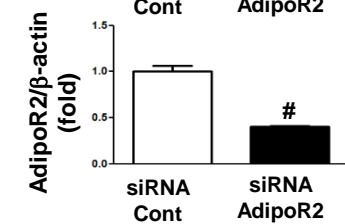

Supplement: Supplementary file 3 — Supplementary Figure S2 [file 41419_2022_4726_MOESM3_ESM.pdf]

**Fig. 2**

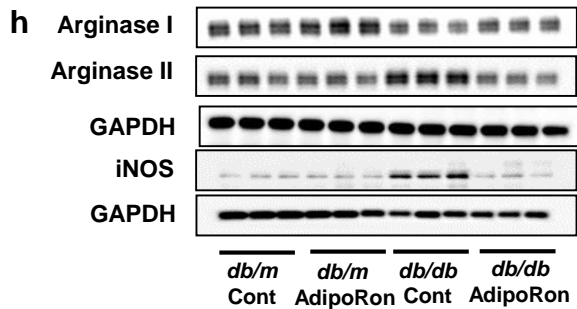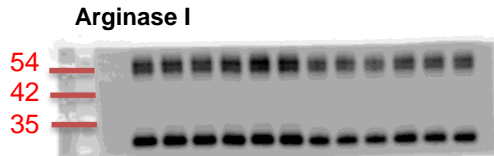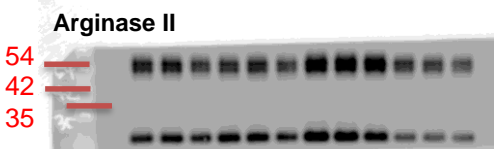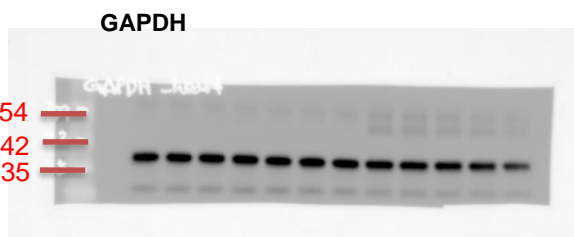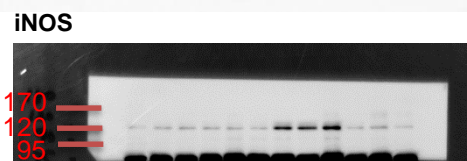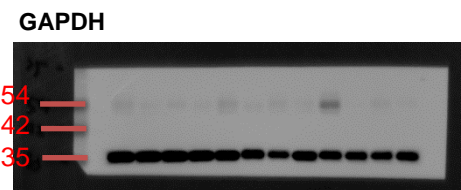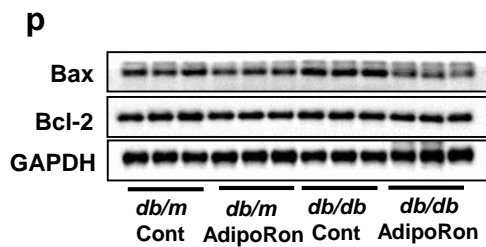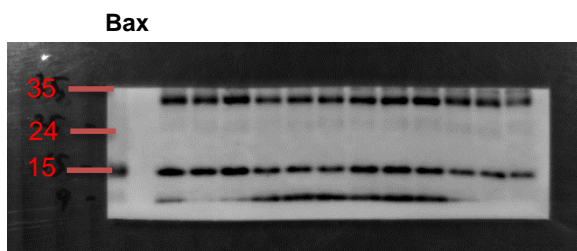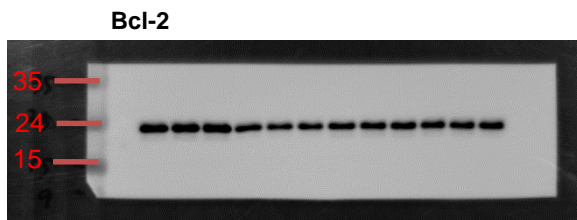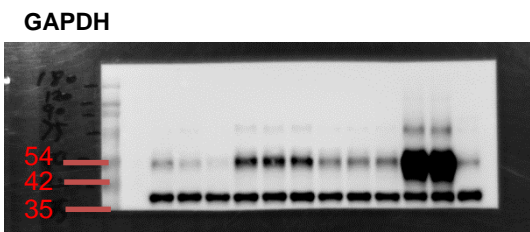

Supplement: Supplementary file 4 — Supplementary Figure S3 [file 41419_2022_4726_MOESM4_ESM.pdf]

Fig. 3

d

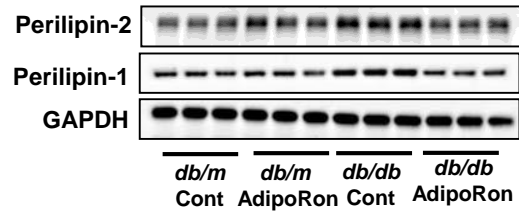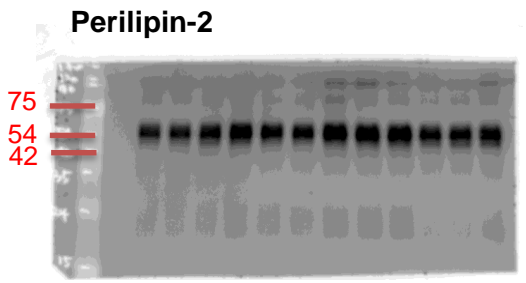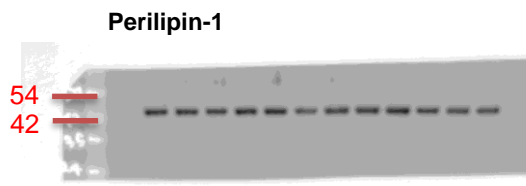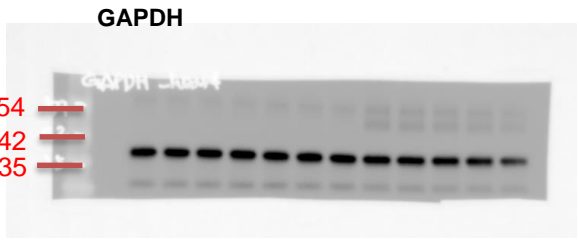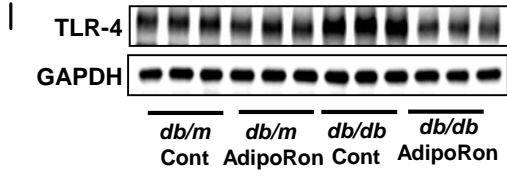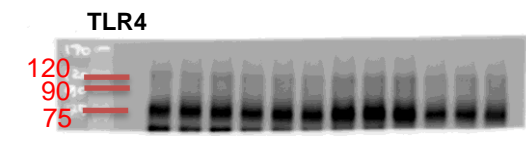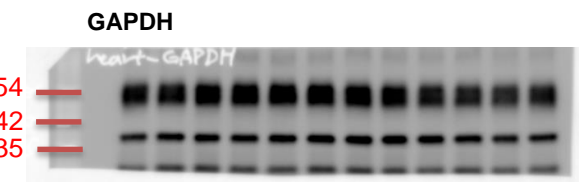

Supplement: Supplementary file 5 — Supplementary Figure S4 [file 41419_2022_4726_MOESM5_ESM.pdf]

Fig. 5

a

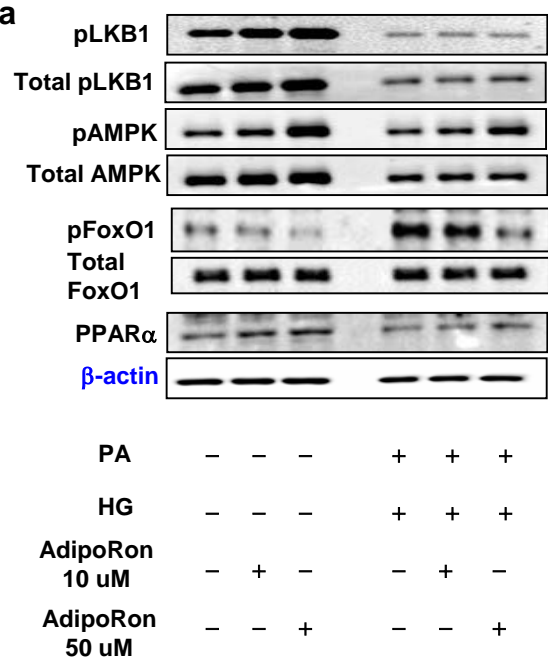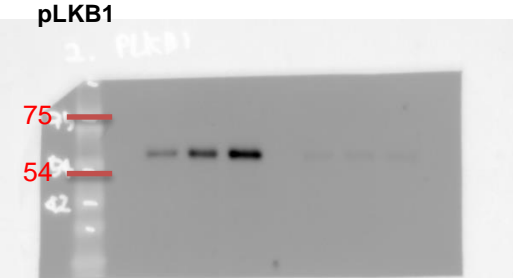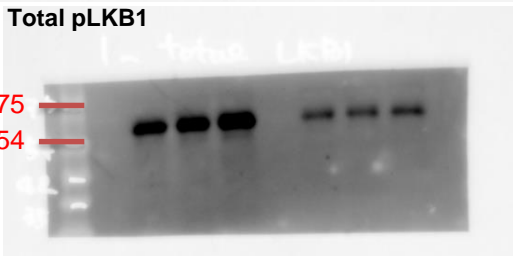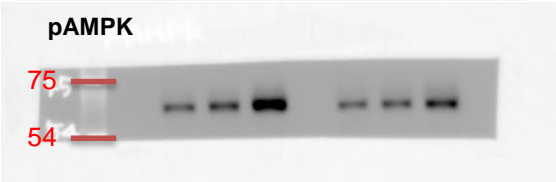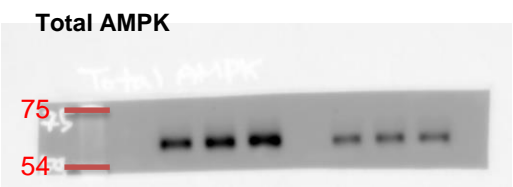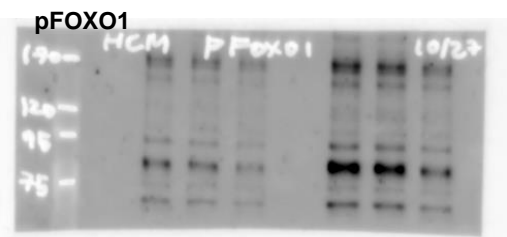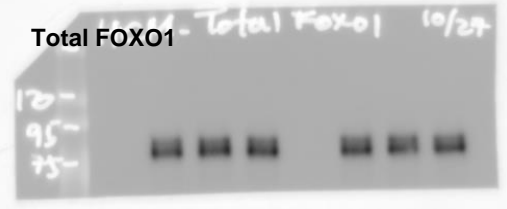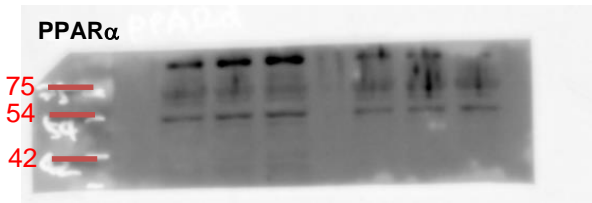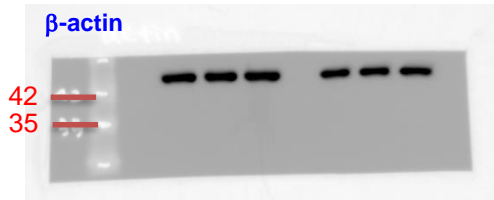

Fig. 5

f

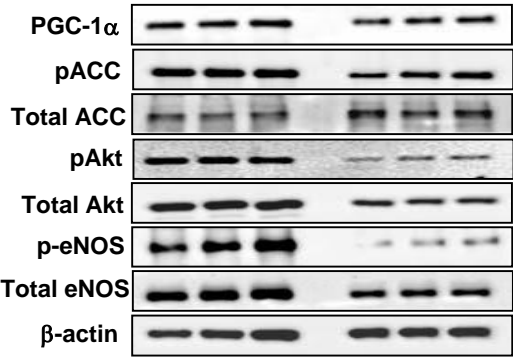

|                   |   |   |   |   |   |   |
|-------------------|---|---|---|---|---|---|
| PA                | - | - | - | + | + | + |
| HG                | - | - | - | + | + | + |
| AdipoRon<br>10 uM | - | + | - | - | + | - |
| AdipoRon<br>50 uM | - | - | + | - | - | + |

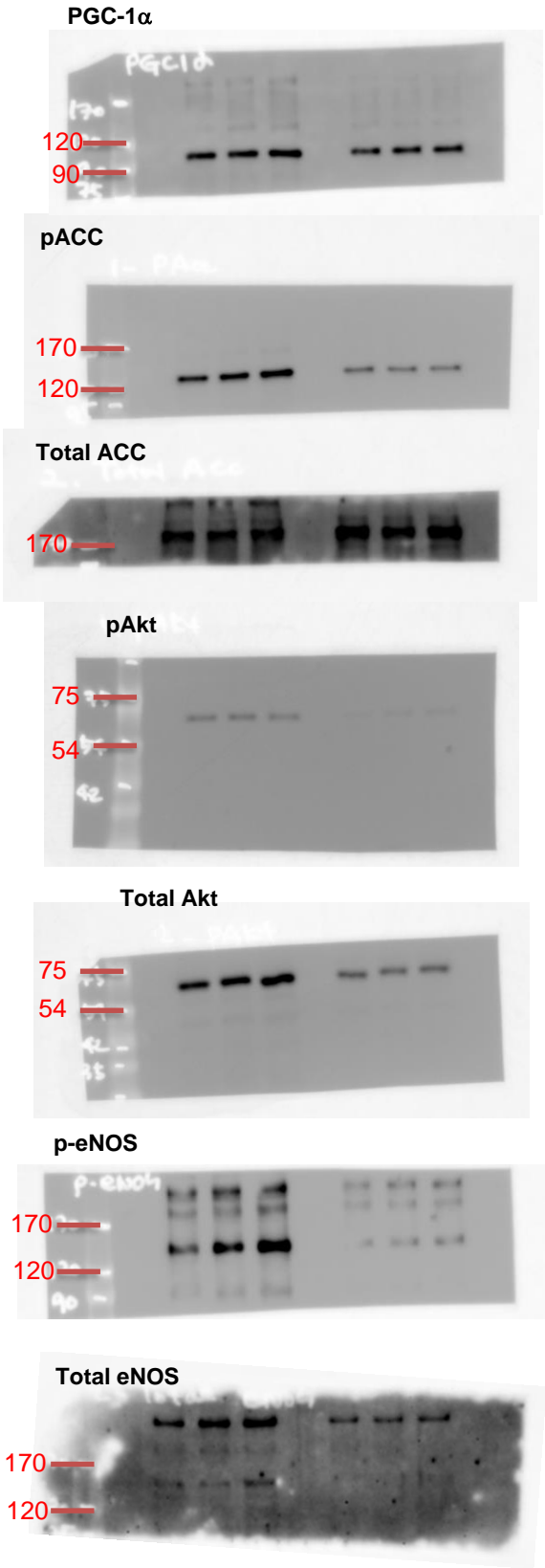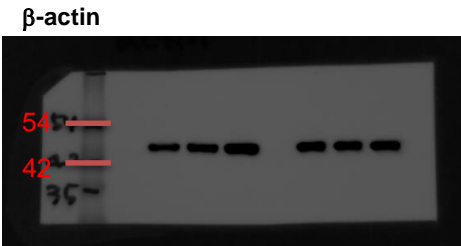

Supplement: Supplementary file 7 — Supplementary Figure S6 [file 41419_2022_4726_MOESM7_ESM.pdf]

**Fig. 6**  
**i**

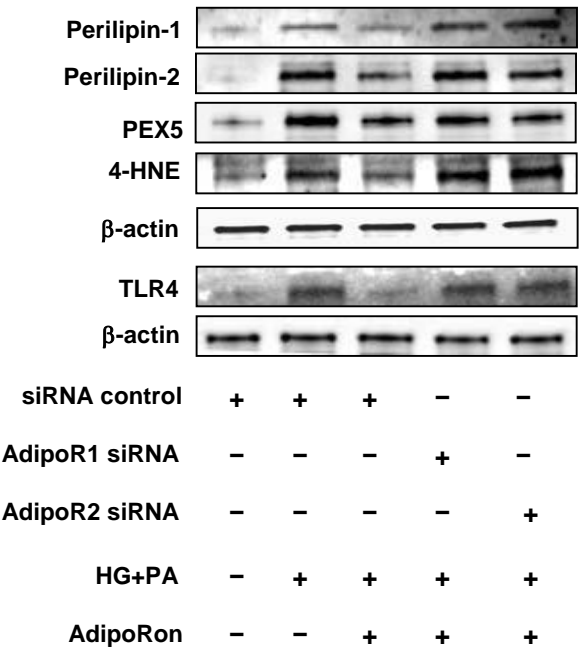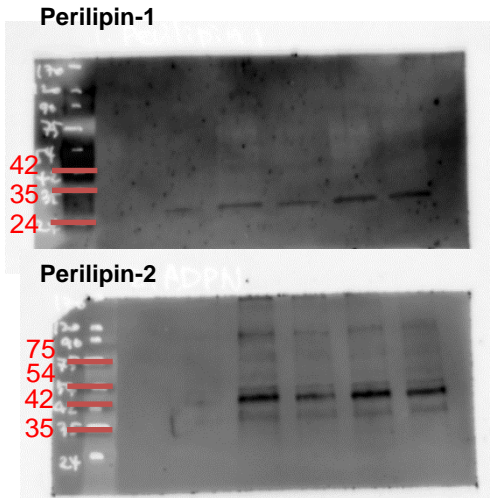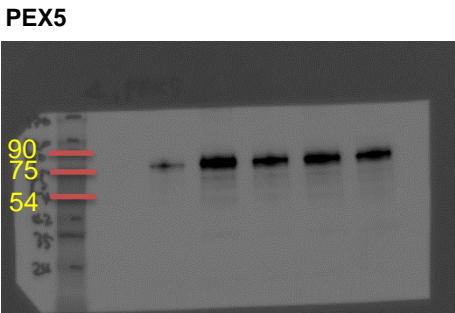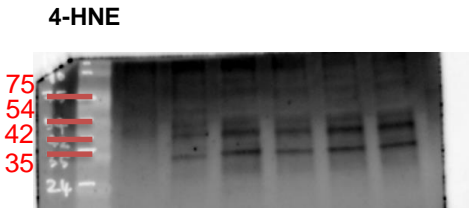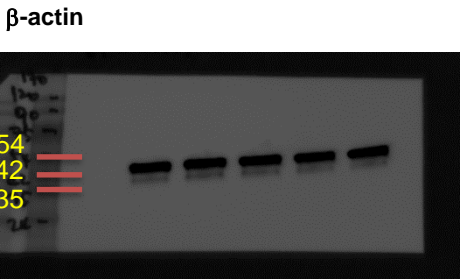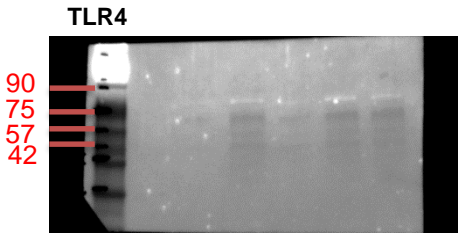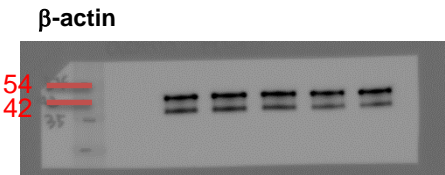

Supplement: Supplementary file 8 — Supplementary Figure S7 [file 41419_2022_4726_MOESM8_ESM.pdf]

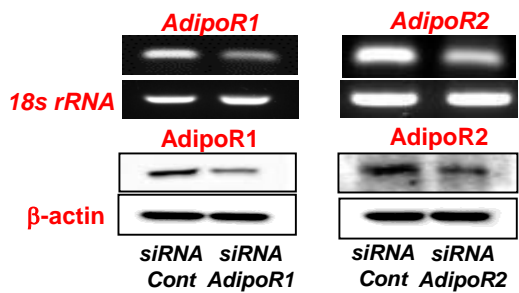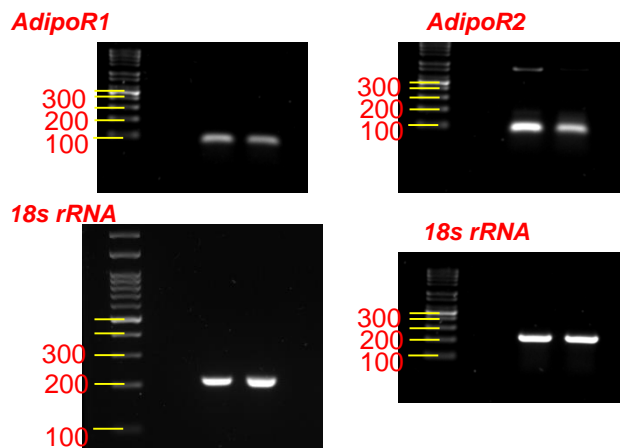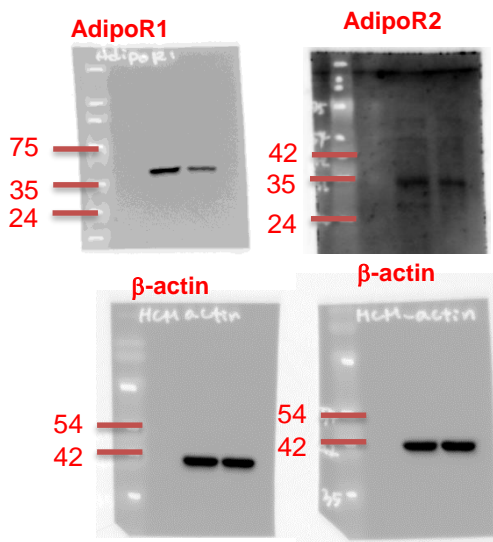

Supplement: Supplementary file 10 — Supplementary Figure S8 [file 41419_2022_4726_MOESM10_ESM.pdf]
