# Supplementary material for: Adiponectin receptor agonist ameliorates cardiac lipotoxicity via enhancing ceramide metabolism in type 2 diabetic mice
Source: Cell Death Dis. 2022 Mar 30;13(3):282. doi: 10.1038/s41419-022-04726-8 (PMC8964809; doi:10.1038/s41419-022-04726-8)
Supplement: Supplementary file 6 — Supplementary Figure S5 [file 41419_2022_4726_MOESM6_ESM.pdf]

Fig. 4

a

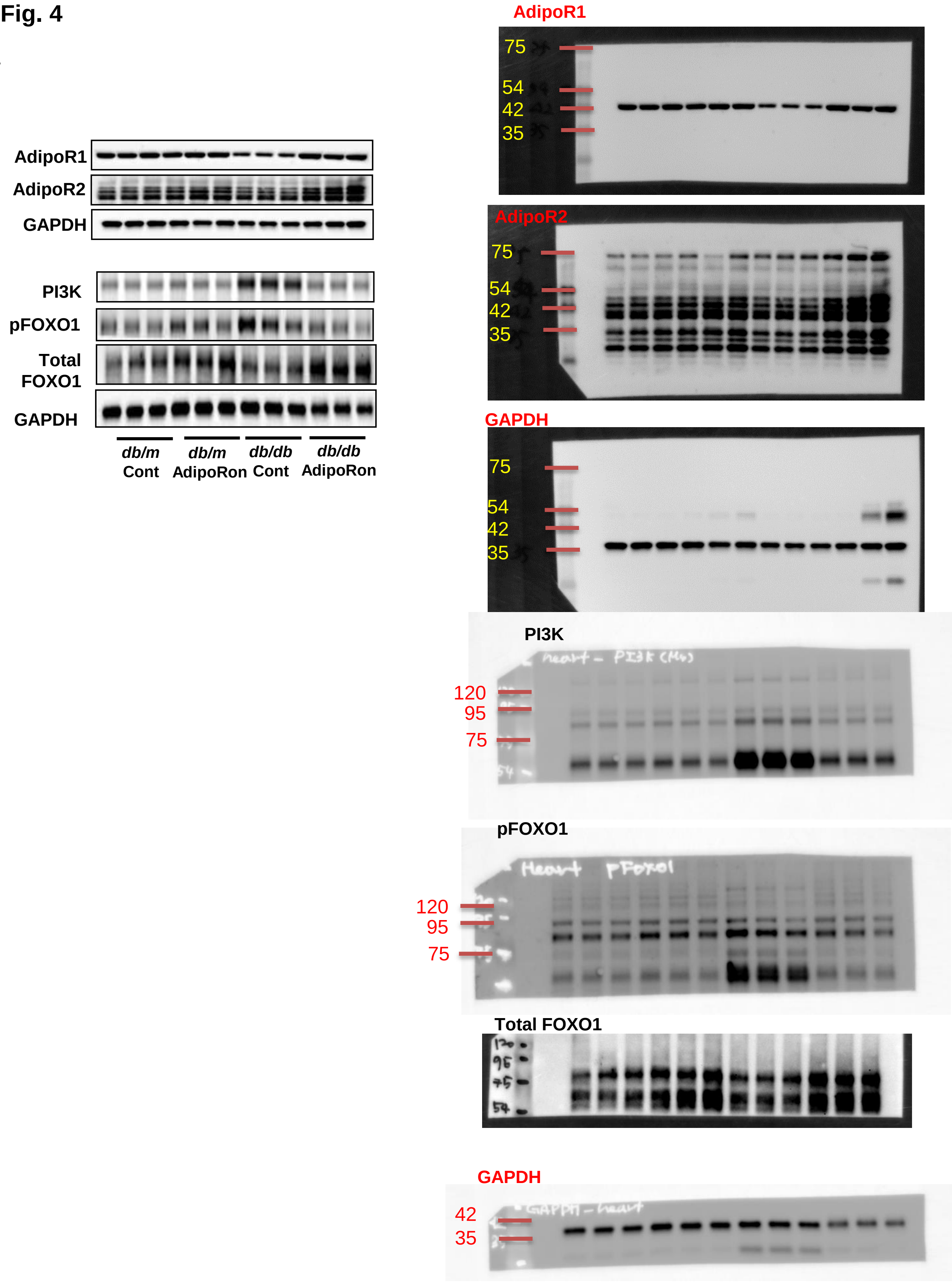

AdipoR1
AdipoR2
GAPDH
PI3K
pFOXO1
Total FOXO1
GAPDH
db/m Cont
db/m AdipoRon
db/db Cont
db/db AdipoRon
AdipoR1
75
54
42
35
AdipoR2
75
54
42
35
GAPDH
75
54
42
35
PI3K
120
95
75
pFOXO1
120
95
75
Total FOXO1
GAPDH
42
35

Fig. 4

g

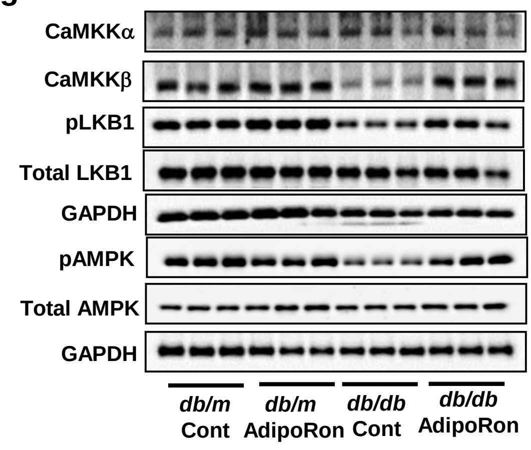

CaMKKα
CaMKKβ
pLKB1
Total LKB1
GAPDH
pAMPK
Total AMPK
GAPDH
db/m
Cont
db/m
AdipoRon
db/db
Cont
db/db
AdipoRon

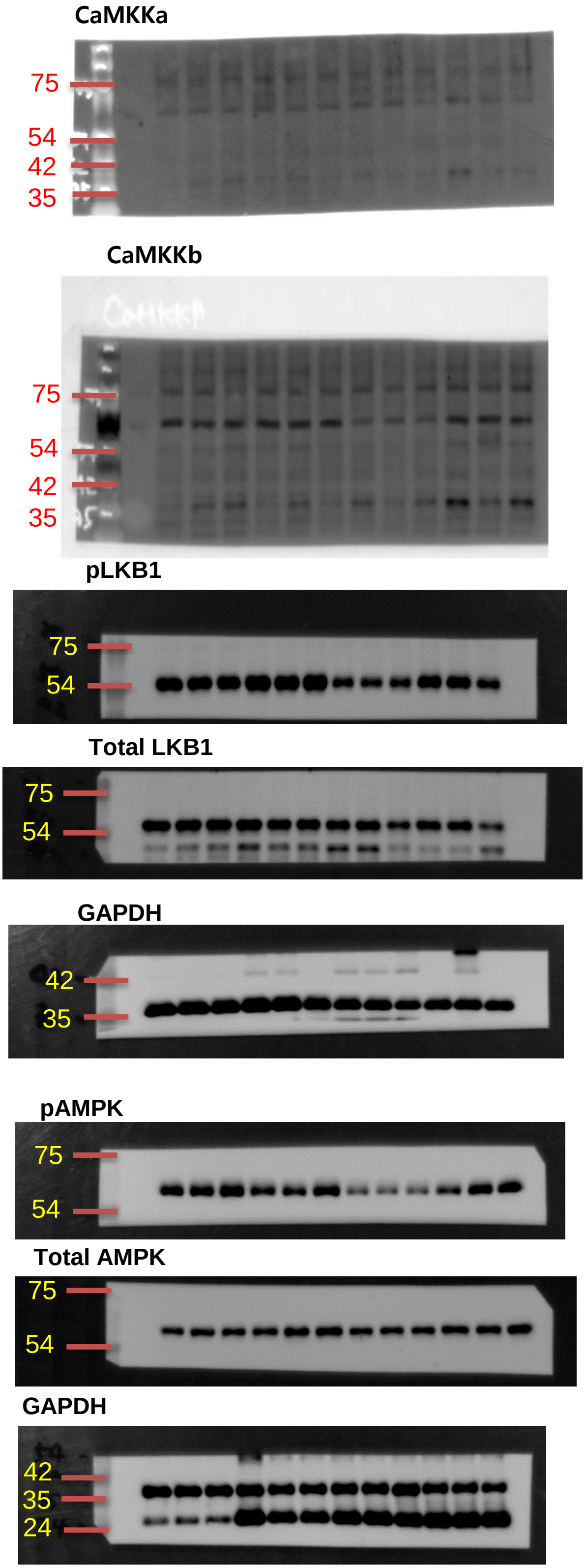

CaMKKa
75
54
42
35
CaMKKb
75
54
42
35
pLKB1
75
54
Total LKB1
75
54
GAPDH
42
35
pAMPK
75
54
Total AMPK
75
54
GAPDH
42
35
24

Fig. 4

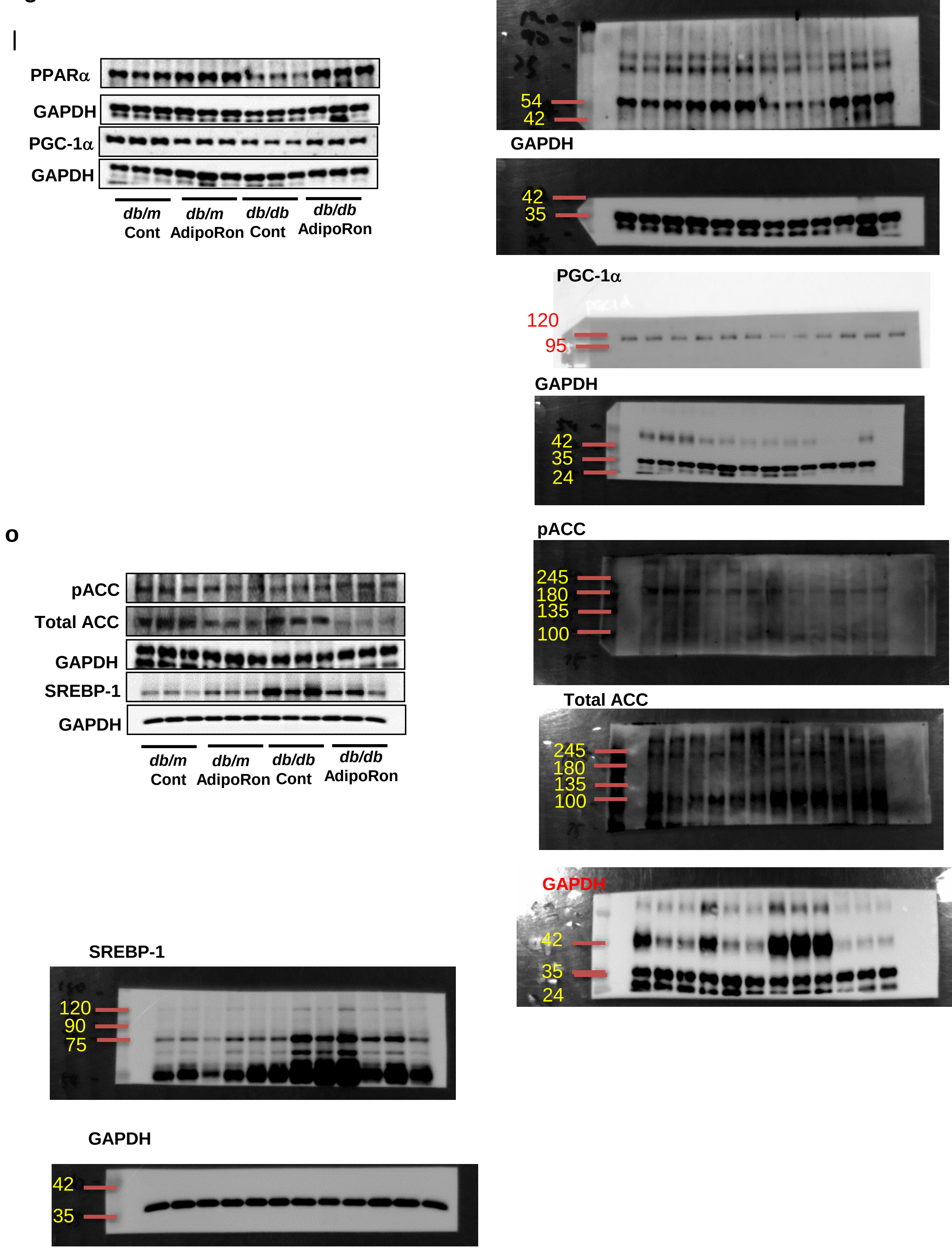
l
PPARα
GAPDH
PGC-1α
GAPDH
db/m Cont
db/m AdipoRon
db/db Cont
db/db AdipoRon
PPARα
54
42
GAPDH
42
35
PGC-1α
120
95
GAPDH
42
35
24
o
pACC
Total ACC
GAPDH
SREBP-1
GAPDH
db/m Cont
db/m AdipoRon
db/db Cont
db/db AdipoRon
pACC
245
180
135
100
Total ACC
245
180
135
100
GAPDH
42
35
24
SREBP-1
120
90
75
GAPDH
42
35

r

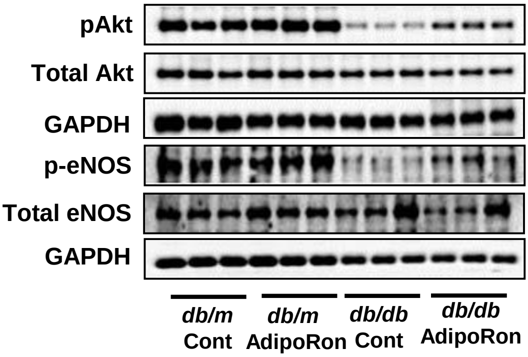
pAkt
Total Akt
GAPDH
p-eNOS
Total eNOS
GAPDH
db/m Cont
db/m AdipoRon
db/db Cont
db/db AdipoRon

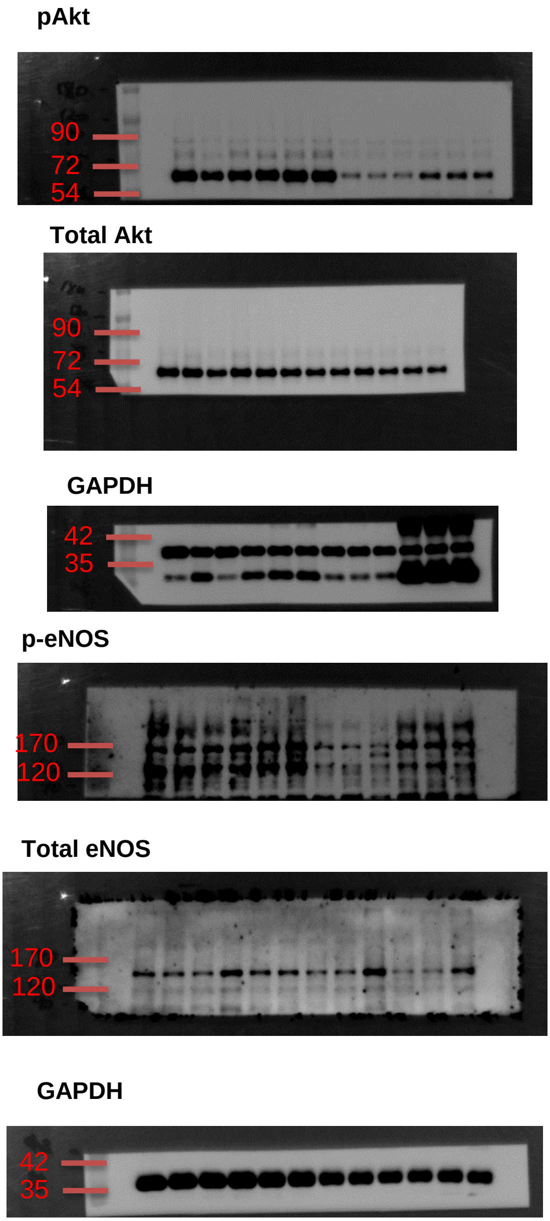
pAkt
90
72
54
Total Akt
90
72
54
GAPDH
42
35
p-eNOS
170
120
Total eNOS
170
120
GAPDH
42
35
