# Supplementary material for: Adiponectin receptor agonist ameliorates cardiac lipotoxicity via enhancing ceramide metabolism in type 2 diabetic mice
Source: Cell Death Dis. 2022 Mar 30;13(3):282. doi: 10.1038/s41419-022-04726-8 (PMC8964809; doi:10.1038/s41419-022-04726-8)
Supplement: Supplementary file 9 — Supplementary Figure S8 [file 41419_2022_4726_MOESM9_ESM.pdf]

Fig. 7

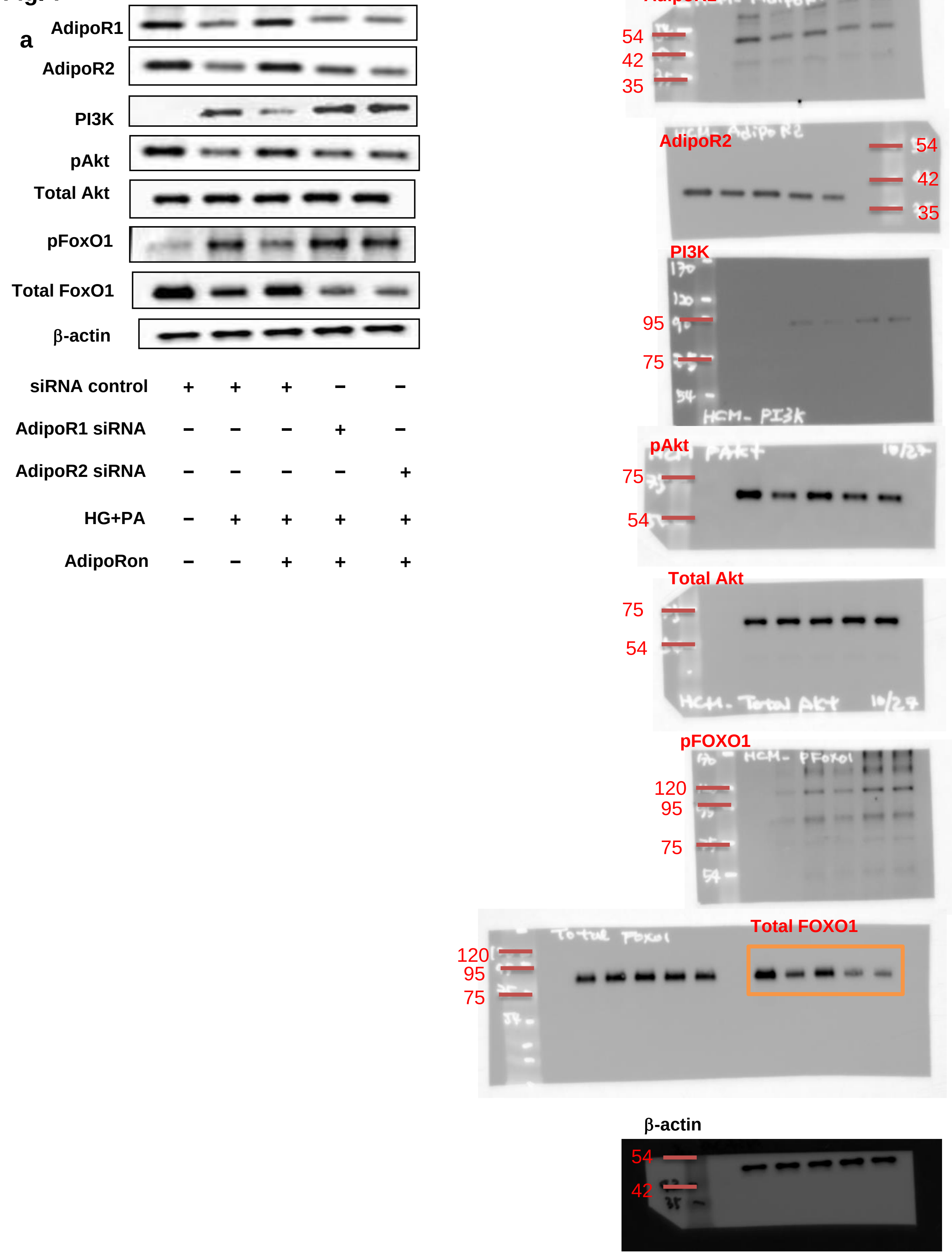
a
AdipoR1
AdipoR2
PI3K
pAkt
Total Akt
pFoxO1
Total FoxO1
β-actin
siRNA control + + + − −
AdipoR1 siRNA − − − + −
AdipoR2 siRNA − − − − +
HG+PA − + + + +
AdipoRon − − + + +
AdipoR1
54
42
35
AdipoR2
54
42
35
PI3K
95
75
pAkt
75
54
Total Akt
75
54
pFOXO1
120
95
75
Total FOXO1
120
95
75
β-actin
54
42

j

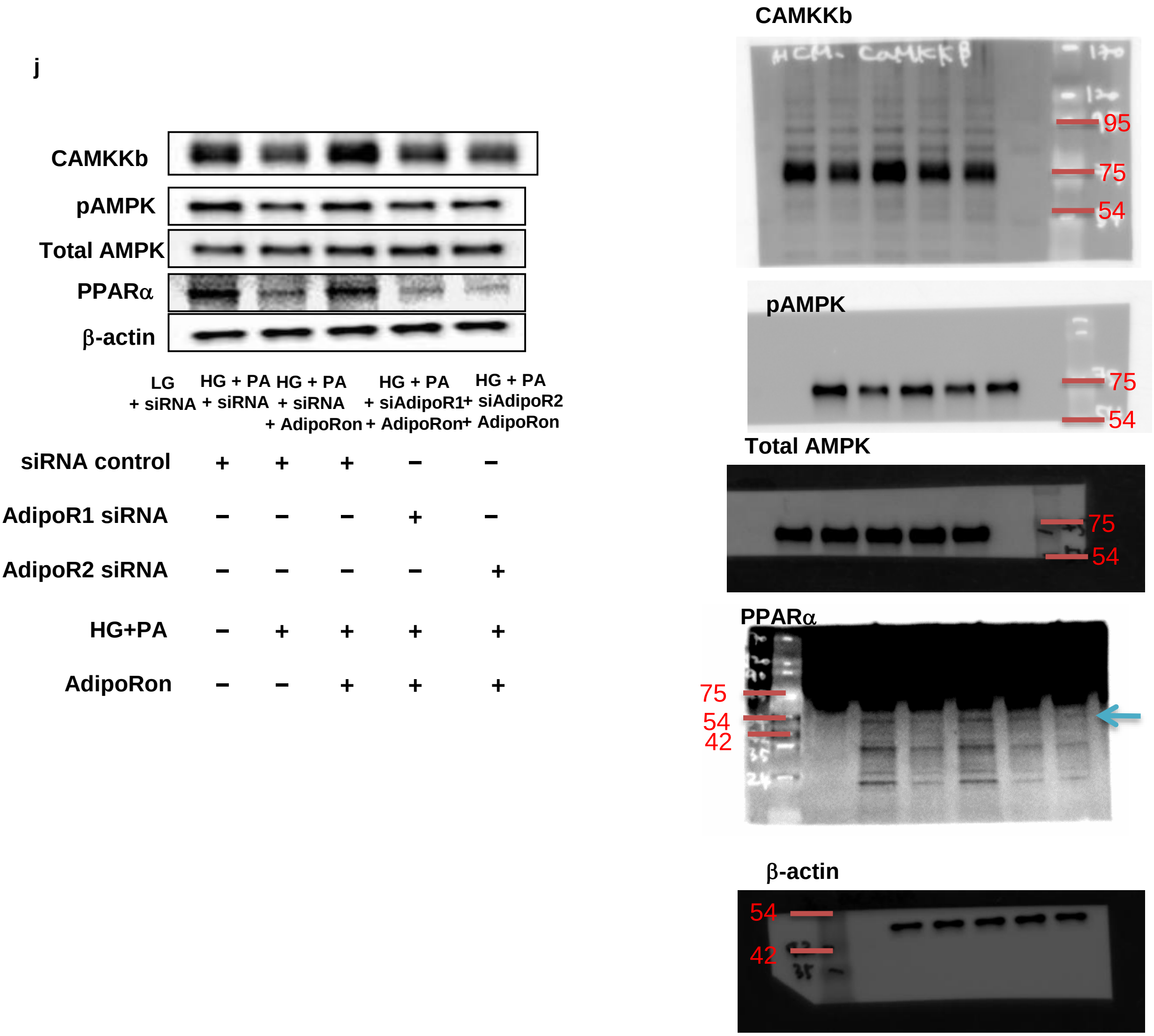
CAMKKb
pAMPK
Total AMPK
PPARα
β-actin
LG + siRNA
HG + PA + siRNA
HG + PA + siRNA + AdipoRon
HG + PA + siAdipoR1 + AdipoRon
HG + PA + siAdipoR2 + AdipoRon
siRNA control + + + − −
AdipoR1 siRNA − − − + −
AdipoR2 siRNA − − − − +
HG+PA − + + + +
AdipoRon − − + + +
CAMKKb
95
75
54
pAMPK
75
54
Total AMPK
75
54
PPARα
75
54
42
β-actin
54
42
